# Bone Marrow Mesenchymal Stromal Cell Osteogenesis is driven by Paracrine signals from Regulatory T Cell

**DOI:** 10.1007/s12015-025-11015-2

**Published:** 2025-11-10

**Authors:** Eylem Baysal, Niyaz Al-Sharabi, Kamal Mustafa, Daniela E. Costea, Meadhbh Brennan, Salwa Suliman

**Affiliations:** 1 Center of Translational Oral Research (TOR)—Tissue Engineering Group, Department of Clinical Dentistry, Faculty of Medicine, University of Bergen, Bergen, Norway; 2 The Gade Laboratory for Pathology and Center for Cancer Biomarkers (CCBIO), Department of Clinical Medicine, University of Bergen, Bergen, Norway; 3 Department of Pathology, Haukeland University Hospital, Bergen, Norway; 4 Regenerative Medicine Institute, School of Medicine, and Biomedical Engineering, School of Engineering, University of Galway, Galway, Ireland

**Keywords:** Secretome, Bone regeneration, Immunomodulation, Stem cells

## Abstract

Bone regeneration is a dynamic process regulated by the interplay between the immune and skeletal systems. Regulatory T cells (Treg), a specialized subset of CD4^+^ T cells, play a crucial role in immunomodulation and bone regeneration by regulating the immune response and interacting with progenitor cells. However, the specific mechanisms through which Treg influence the osteogenic differentiation of bone marrow stromal cells (BMSC) remain unexplored. Treg were isolated from six healthy donors, expanded for 13 days, and starved for 24 h to collect Treg-conditioned media (Treg-CM). BMSC obtained from three different healthy donors were treated with Treg-CM at an optimized concentration (50 µg/mL) to assess its impact on BMSC metabolic activity, migration, and osteogenic differentiation. Label free proteomics and cytokine profiling were conducted to identify unique proteins and immunomodulatory factors in Treg-CM. The secretory cytokines of BMSC treated with Treg-CM were also analyzed. Treg-CM enhances BMSC osteogenic differentiation by upregulating the expression of key osteoblast-specific genes, increasing ALP activity, and facilitating calcium deposition. Proteomics identified unique proteins in Treg-CM that regulate cytoskeletal dynamics, metabolic processes and mRNA regulation, highlighting a complex mechanism underlying Treg-CM effects. Cytokine profiling provided key immune modulators in Treg-CM that regulate osteogenesis. Furthermore, elevated levels of MIP-1α and G-CSF were secreted by BMSC treated with Treg-CM further supporting its role in immune-mediated osteogenesis. Our findings reveal that Treg-CM enhances not only osteogenesis in vitro but also fosters a pro-regenerative microenvironment. This highlights its potential as a cell-free strategy for enhancing stem-cell based osteogenesis.

**Graphical Abstract:**

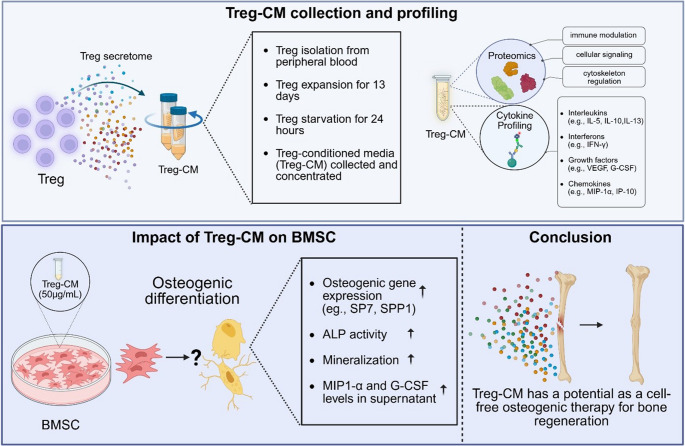

**Supplementary Information:**

The online version contains supplementary material available at 10.1007/s12015-025-11015-2.

## Introduction

Bone regeneration is a complex process entailing a well-orchestrated series of biological events involving various cell types and molecular signals [1]. Immune cells play a crucial role in this process by regulating the activity of osteoblasts, osteoclasts, and bone marrow derived mesenchymal stromal/stem cells (BMSC) through signaling cascades and the release of bioactive factors, which facilitate intracellular communication for effective bone remodeling [2]. Notably, BMSC have been extensively studied in preclinical and clinical trials for their immunomodulatory and bone regeneration capacity, and an efficient bone repair depends on the crosstalk between implanted BMSC and recipient immune cell subsets [3–5]. Most research about the role of immunity in bone regeneration has focused on the innate component of the immune response. Recent, co-culture studies of BMSC with macrophage and T cells reveal a cross-talk and interdependent modulation that leads to enhanced BMSC osteogenesis [6]. However, emerging evidence shows that T lymphocytes are particularly critical for maintaining bone homeostasis, survival, and function of osteoclasts and osteoblasts [7]. Delayed bone fracture healing was significantly associated with a high ratio of terminally differentiated CD8^+^ T cells in peripheral blood and fracture hematoma [8]. Additionally, substantial T cell infiltration has been observed in the fracture callus around day 14 after injury, especially in newly forming bone areas [9]. T cell subsets differentially regulate BMSC-mediated bone formation in vivo, as shown by infusing different T cells into nude mice; proinflammatory T cells inhibited bone regeneration, whereas regulatory T cells (Treg), a specialized subpopulation of CD4^+^ T cells, promoted it [10].

Research has predominantly focused on the inhibitory effects of Treg on osteoclasts as a mechanism of action in promoting bone formation. For instance, Treg suppress osteoclast differentiation from peripheral blood mononuclear cells through a cytokine-dependent mechanisms [11]. Equally, Treg also inhibit osteoclast differentiation through direct cell-to-cell contact, primarily mediated by cytotoxic T lymphocyte antigen 4 [12]. Furthermore, adoptive transfer of Treg from healthy mice into osteogenesis imperfecta mice reduces T cell activation and effector cytokine secretion, leading to improved bone structure [7]. Consistently, Treg conditioned medium (Treg-CM) from healthy mice reduced mouse osteoclast numbers and increased alkaline phosphatase activity and extracellular matrix mineralization by osteoblasts in vitro [7]. These findings suggest that Treg and their secretome regulate bone regeneration by modulating both activity of osteoclasts and osteoblasts.

Treg are key modulators of the immune system and self-tolerance maintainers by suppressing the immune response and contributing to tissue repair and regeneration by directing both innate and adaptive immune system, as well as through interaction with non-immune cells [13]. It has been reported that Treg promote new bone formation by secreting IL-10 to suppress Th17 functionality [14]. More recently, a specific CCR8^+^ Treg subset has been shown to infiltrate bone injury sites and promote skeletal stem cells osteogenesis through progranulin secretion [15]. Similarly, local administration of CD4^+^CD25^hi^FOXP3^+^ Treg has been shown to promote tissue healing in musculoskeletal defects in vivo in mice via IL-10 secretion [16]. These studies highlight the important role of Treg and their secretome in bone regeneration, however the specific interaction with bone progenitiors, BMSC, remains limitedly explored.

 Regarding the cross-talk of Treg and bone progenitors, studies have shown that Treg infusion improves BMSC-mediated bone regeneration in vivo [10]. Reciprocally, BMSC have been shown to modulate Treg and promote bone regeneration as evidenced by reduced IFN-γ and TNF-α levels and increased splenic Treg following systemic BMSC infusion, enhancing BMSC-scaffold bone regeneration [17]. BMSC-Treg co-culture studies revealed that BMSC induce Foxp3 expression and suppressive funtionality in Treg through mitochondrial transfer or CD80-mediated contact-dependent interactions [18, 19].

 Despite growing interest in the immunomodulatory interactions between BMSC and Treg, existing studies have primarily focused on how BMSC influence Treg function or indirect Treg effects on bone regeneration, leaving the direct impact of Treg or their secretome on BMSC osteogenesis largely unexplored. Only one study has investigated co-culture of BMSC with induced Treg (differentiated from naïve CD4⁺ T cells), showing increased alkaline phosphatase (ALP) activity. However, this effect effect was comparable to that seen with naïve CD4⁺ T cells. Moreover, in this same study, Treg-CM showed only modest, concentration-dependent effects on ALP activity, assessed only at a single time point [20]. These findings suggest a limited role of induced Treg in BMSC osteogenesis and underscore the need for more comprehensive studies.

Therefore, this study addresses the gap by aiming to evaluate the effects of natural Treg secretome on BMSC osteogenesis. Treg-CM was standardized based on protein concentration to ensure consistent and accurate dosing for BMSC. By providing detailed insights into the components of the Treg secretome and evaluating its impact on BMSC osteogenesis using multiple molecular and functional parameters, our study offers a novel perspective on Treg-BMSC interactions.

## Materials and Methods

### Isolation and Characterization of Treg and BMSC

Treg were isolated from the peripheral blood of *six* healthy human donors, with informed constent through the Blood Bank, Bergen, Norway, using the MACSxpress^®^ Whole Blood Isolation Kit (Miltenyi Biotec, Germany). Treg were expanded using CD3/CD28 MACSiBead™ (Miltenyi Biotec), and characterized using the human Treg detection kit (Miltenyi Biotec) according to manufacturer’s instructions.

Human BMSC were isolated from bone marrow harvested from the iliac crest of three healthy donors following informed constent, with approval from the Regional Committees for Medical and Health Research Ethics (REK) in Norway (2020/7199/REK sør-øst C). The donors for Treg and BMSC are unmatched. The bone marrow was diluted with standard growth medium (GM), which is Dulbecco’s Modified Eagle’s medium (DMEM) (Invitrogen, USA) supplemented with 10% (v/v) fetal bovine serum (FBS) (GE Healthcare, USA) and 1% (v/v) penicillin/streptomycin (P/S) (GE Healthcare), and filtered through a 70 μm cell strainer before centrifugation at 600 × g for 10 min. Cells were cultured in GM with media changes every third day. BMSC from passage 3–5 were used in experiments after surface marker characterization by flow cytometry. Briefly, cells were incubated with anti-CD73, anti-CD90, anti-CD105, anti-CD34, anti-CD45, and anti-HLA-DR (BD Biosciences, CA) following manufacturer’s guidelines. Briefly, BMSC (4 × 10^5^) were suspended in 400 µL of Dulbecco’s Phosphate-Buffered Saline (DPBS) (Thermo Fisher Scientific, USA) and centrifuged at 200 × g for 5 min at 4 °C. Subsequently, 20 µL of blocking reagent [0.5% bovine serum albumin (BSA) (Sigma-Aldrich, USA) in DPBS] was added, followed by incubation for 10 min at room temperature. Fluorescent monoclonal antibodies were added and incubated in the dark for 30 min at 4 °C. Samples were analyzed using a BD Accuri C6 flow cytometer and analyzed using FlowJo V10.6.2 [21].

For multilineage characterization, BMSC were seeded at a density of 3 × 10^3^ cells/cm^2^. After 24 h, cells were cultured in osteogenic media (OM) (DMEM, 10% FBS, 1% p/s supplemented with 173 µM L-ascorbic acid 2-phosphate, 10 nM dexamethasone, 10 mM β glycerophosphate; all from Sigma-Aldrich) for 7, 14 and 21 days. For adipogenic differentiation, BMSC were seeded at 7 × 10^3^ cells/cm^2^. After 24 h, cells were cultured in adipogenic differentiation medium (DMEM, 10% FBS, 1% p/s supplemented with 100 nM dexamethasone, 10 µg/mL insulin, 0.2 mM indomethacin, 0.5 mM 3-Isobutyl-1-methylxanthine; all from Sigma-Aldrich) for 14 days. Control cells were maintained in GM. Lipid droplets were stained with Oil Red O staining and osteogenic differentiation was assessed on day 7 by ALP staining, and on days 14 and 21 by Alizarin Red staining, as previously described [21].

### Optimization of Culture Conditions for Treg and BMSC

To optimize the culture media and duration for Treg expansion, Treg from the same donor were cultured for either 14 or 21 days under two conditions; TexMACS medium (Miltenyi Biotec) or Roswell Park Memorial Institute (RPMI) 1640 medium (Gibco, Thermo Fisher Scientific) supplemented with 5% AB serum (Sigma-Aldrich) and 500 U/mL IL-2 (Miltenyi Biotec). Following the respective culture periods, Treg were starved for 24 h and characterized as detailed in Supplementary Table S1. Treg viability was assessed using the LIVE/DEAD™ Fixable Far Red Dead Cell Stain Kit (Invitrogen). To optimize the culture conditions and evaluate the impact of TexMACS media on BMSC; BMSC from three different donors were cultured in a gradient of TexMACS percentages before being tested for metabolic activity, morphology and stemness. To assess the impact of TexMACS on BMSC metabolic activity, cells were culture in 25% TexMACS, 50% TexMACS, or 100% TexMACS medium, each diluted with DMEM and supplemented with 10% FBS and 1% p/s. BMSC metabolic activity was assessed on days 1, 4, and 7 using the PrestoBlue™ Cell Viability Reagent (Invitrogen), following the manufacturer’s protocol. Briefly, PrestoBlue solution was diluted in GM (1:10) and added directly to all wells. After 30 min incubation at 37 °C with 5% CO_2_, fluorescence intentisty was measured using a Varioskan LUX Multimode Microplate Reader (Thermo Fisher Scientific). Fluorescence values were divided by the mean value of the GM control group, multiplied by 100, and expressed as percentages.

For morphological and stemness assessment, BMSC were seeded (3 × 10^3^ cells/cm²) on chamber slides (µ-Slide 8 Well, ibidi GmbH, Germany). After 24 h, the cells were treated with 25%, 50%, or 100% TexMACS media for 7 days, with media changed twice/week. Cells were fixed with 4% paraformaldehyde (Sigma-Aldrich) for 10 min, permeabilized (Invitrogen) for 30 min, and blocked with 3% BSA and 10% Normal Goat Serum (Thermo Fisher Scientific) in DPBS for 1 h. Cells were then incubated overnight at 4 °C with anti-STRO-1 antibody (1:150) (Santa Cruz Biotechnology, USA) in 3% BSA/DPBS, and followed by Alexa Fluor™ 488 secondary antibody (1:200) (Thermo Fisher Scientific) in DPBS for 1 h. Nuclei and cytoskeleton was stained with DAPI (1:1000) (Thermo Fisher Scientific) and Phalloidin Atto 565 (1:500) (Sigma-Aldrich) in DPBS for 1 h. Images were taken by Dragonfly 505 confocal microscope (Andor Technologies Inc, Northern Ireland).

### Collection of Treg-CM

Treg were isolated from six healthy donors and expanded for 13 days with TexMACS medium (Miltenyi Biotec) supplemented with 5% AB serum and 500 U/mL IL-2. On day 13, cells were centrifuged at 300 × g for 5 min and washed once with DPBS before being seeded serum-free with TexMACS and 50 U/mL IL-2 only. After 24 h, culture media was collected and centrifuged at 300 × g for 5 min at 4 °C followed by the centrifugation of the supernatant at 2000 × g for 20 min at 4 °C to remove cell debris. Thereafter, the final supernatant was filtered through a 0.2 μm filter, and is referred to as Treg-CM.

Treg from all six donors was characterized as shown in Supplementary Table S2, before Treg-CM was pooled and stored at −80 °C. In parallel, a control cell-free culture media supplemented with only 50 U/mL IL-2 was also incubated for 24 h and collected, treated in the same way as Treg-CM and referred to as Non-CM. A portion of the pooled Treg-CM and Non-CM was concentrated using Amicon ultrafilters (MWCO = 30 kDa; Merck Millipore, USA) and protein concentrations quantified using BCA assay (Thermo Fisher Scientific) before being aliquoted and frozen at −80 °C for use in experiments. Non-concentrated Treg-CM and Non-CM were used in percentages for initial assessments, with details provided in the Supplementary Methods section.

### Selection of Treg-CM Concentration

BMSC (3 × 10^3^ cells/cm²) were seeded and allowed to adhere overnight, then treated with Treg-CM or Non-CM (50, 100, or 250 µg/mL) in GM, replenished twice weekly. BMSC metabolic activity was assessed on days 1, 4, and 7 using the PrestoBlue™ Cell Viability Reagent (Invitrogen), following the manufacturer’s protocol and 50 µg/mL was identified as the optimal concentration for further experiments.

### Impact of Treg-CM on BMSC Morphology and Stemness

BMSC (3 × 10^3^ cells/cm²) were seeded, and after 24 h cells were cultured with 50 µg/mL Treg-CM or Non-CM in GM for 4 days. After the treatment period, cells were stained with anti-STRO-1 and phalloidin to assess morphology and stemness as described in Section Optimization of Culture Conditions for Treg and BMSC .

### Impact of Treg-CM on BMSC Migration Potential

BMSC (3 × 10^5^ cells/mL) were seeded into culture-inserts (Ibidi culture-insert 2 well, ibidi GmbH) in GM. After 24 h, cells were incubated with Mitomycin C-ReadyMade Solution (20 µg/mL; Merck, Germany) for 2 h at 37 °C in 5% CO_2_ to inhibit cell proliferation. The inserts were then removed and non-adherent cells washed away with DPBS, before treating BMSC with Treg-CM or Non-CM (50 µg/mL) in GM. Images were captured after 0, 24, 48 and 72 h using an inverted microscope, and after 72 h, cells were stained with 0.5% crystal violet solution (Merck Millipore, USA) for 10 min. The decrease in average distances of lines drawn along cell fronts was analyzed using ImageJ software [22]. Migration area was calculated using the formula: migration area (%) = (A0 - Af)/A0 × 100, where A0 is the initial wound area (t = 0 h) and Af is the residual wound area at t = 24, 48–72 h respectively [23].

### Impact of Treg-CM on BMSC Osteogenic Differentiation at mRNA Level

BMSC (3 × 10³ cells/cm²) were cultured in GM for 24 h, then treated with Treg-CM or Non-CM (50 µg/mL) in OM for 10 days, with media replenished twice weekly. Total RNA was isolated from cells after 4, 7 and 10 days using RNeasy Kit^®^ Mini Kit (Qiagen, Germany), following the manufacturer’s instructions. RNA (300 ng) was converted into cDNA using a High-Capacity cDNA Reverse Transcription Kit (Applied Biosystems, USA) following the manufacturer’s protocol. Real-time quantitative polymerase chain reaction (RT-qPCR) was performed using TaqMan Fast Universal PCR Master Mix (Applied Biosystems) on the StepOne™ Real-Time PCR System (Applied Biosystems).

The expressions of key osteogenesis-related genes, including runt-related *RUNX2*, *SP7* known as osterix, *COL1α2*, *TGFB1*, *SPP1* known as osteopontin, *BGLAP* known as osteocalcin and *ALP* were assessed (all from Thermo Fisher Scientific) (Supplementary Table S3). Gene expressions were normalized to housekeeping gene *GAPDH* and differential expressions were calculated using the comparative CT method (2^−ΔΔCT^).

### Impact of Treg-CM on BMSC ALP Activity and Matrix Mineralization

BMSC (3 × 10^3^ cells/cm²) was seeded in GM for 24 h before treatment with Treg-CM or Non-CM (50 µg/mL) in OM for 10 days, and media replaced twice per week.

BMSC were stained for ALP activity after 7 days. Cells were fixed with 4% paraformaldehyde, stained with BCIP/NBT Liquid Substrate System (Sigma-Aldrich) following manufacturer’s instructions. The stain was dissolved in cetylpyridinium chloride (Sigma-Aldrich) overnight for quantification at 540 nm absorbance.

ALP enzymatic activity was measured after 4, 7, and 10 days using the Alkaline Phosphatase Yellow (pNPP) Liquid Substrate System for ELISA (Sigma-Aldrich). Cells were lysed with 0.1% Triton- X100 lysis buffer, followed by two freeze-thaw cycles at −80 °C. Equal amounts of the lysate and liquid substrate were then incubated for 30 min at 37 °C before absorbance was measured at 405 nm [24]. To normalize ALP activity to cell number, total cell number was quantified by using the PicoGreen assay (Thermo Fisher Scientific). Equal amount of cell lysate and PicoGreen working solution were measured at 485 nm (excitation) and 535 nm (emission). The measured ALP activity was first normalized to cell number and then expressed as fold change relative to cells cultured in Non-CM.

Calcium deposition was assessed after 10 days. Cells were fixed with 4% paraformaldehyde stained with 2% Alizarin red S (ARS) (Sigma-Aldrich) for 30 min [24]. The stain was dissolved in cetylpyridinium chloride 2 h at RT for quantification and measured using a microplate reader at 540 nm absorbance.

### Proteomic Profiling of Treg-CM Using LC–MS/MS

Concentrated Treg-CM or Non-CM were analyzed using liquid chromatography with tandem mass spectrometry (LC–MS/MS) with Label-Free Quantitation to identify the unique proteins in Treg-CM and Non-CM. In brief, approximately 20 µg of protein were digested into tryptic peptides. About 0.5 µg of protein dissolved in 2% acetonitrile and 0.5% formic acid was injected into an Ultimate 3000 RSLC system connected online to an Orbitrap Exploris mass spectrometer equipped with EASY-spray nano-electrospray ion source (all from Thermo Scientific).

### Cytokine Analysis Using Multiplex fluorescent-based Immunoassay

The concentrations of 27 cytokines/chemokines (Supplementary Table S4) were analyzed in Treg-CM, Non-CM, as well as from culture supernatant from BMSC treated with either Treg-CM or Non-CM or OM alone at day 7 or day 0 (baseline). For cytokines assessed in BMSC lysate at day 7, cells were lysed in RIPA buffer with 1× Halt protease and phosphatase inhibitors (Thermo Scientific), incubated at 4 °C for 20 min, sonicated, and centrifuged at 16,000 × g for 20 min at 4 °C. Supernatants were used for protein quantification using the BCA Protein Assay Kit (Thermo Scientific). The cytokines/chemokines were detected using Bio-Plex Pro™ Human Cytokine 27-plex Assay (Bio-Rad, USA) on the Luminex platform (Luminex, USA) according to the manufacturer’s instructions. For lysate samples, the results were normalized to the total protein concentration.

### Bioinformatic Analysis

LC–MS/MS raw data were analyzed using Proteome Discoverer (version 2.5.0.400; Thermo Scientific, USA). Protein datasets from Treg-CM or Non-CM were filtered and processed using Perseus (version 2.0.11.1) [25]. Functional enrichment of total proteins in Treg-CM and Non-CM was performed to identify biological processes (BP), cellular components (CC), and molecular functions (MF) relative to the Gene Ontology (GO) databases by using FunRich (version 3.1.3) [26]. Heat maps showing the protein expression levels between Non-CM and Treg-CM were generated with Perseus, while a Venn diagram was created using FunRich to identify the unique proteins in Treg-CM. The protein-protein interaction (PPI) network of unique proteins in Treg-CM was constructed using STRING (version 12.0) with cluster analysis [27]. Cluster analysis divided the network into three primary clusters (k = 3), each subjected to further functional enrichment analysis using FunRich and GO databases. For each cluster, the top five terms in BP, CC, and MF were identified and visualized by using SR Plot [28].

### Statistical Analysis

BMSC from three different donors were characterized and multilineage potential confirmed before further experiments. Experiments assessing Treg-CM in pre-defined concentrations were repeated using BMSC from three independent biological donors, with 3 to 4 technical replicates per donor. Three biological donors were used to confirm experimental robustness and reproducibility. Statistical analyses were performed using Graph Pad Prism 10.0.2 software (GraphPad, USA). Multiple group comparisons were conducted by one-way ANOVA with Tukey’s multiple comparison tests and presented as the mean ± standard error of the mean (SEM). Correlations between cytokine levels (measured in the BMSC culture supernatant at day 7) and osteogenic outcomes (quantified ALP staining at day 7 and ARS staining at day 10) in BMSC treated with 50 µg/mL Treg-CM were assessed using Pearson correlation analysis in RStudio. P values *≤* 0.05 were considered statistically significant.

### Ethical Considerations

Human BMSC were isolated from the iliac crest-derived bone marrow of three healthy donors undergoing standard surgical procedures, following informed consent from donors. The protocol was approved by the Regional Committees for Medical and Health Research Ethics (REK) in Norway (2020/7199/REK sør-øst C). Treg were isolated from the peripheral blood of six healthy donors with informed consent from Blood Bank Bergen, Norway.

## Results

### TexMACS Medium Enhances Treg Expansion and Induces a dose-dependent Limitation on BMSC Growth

Treg were phenotyped within the CD45^⁺^CD4^⁺^CD127^dim/−^ population, with CD25^⁺^Foxp3^⁺^ double positivity **(**Supplementary Fig. 1a). Their viability was assessed using a live/dead assay (Supplementary Fig. 1b) and was analyzed at days 14 and 21 in both RPMI and TexMACS, with and without starvation (Supplementary Fig. 1c). The morphology of Treg cultured in TexMACS and RPMI media was observed at days 4, 7, 14, and 21. By day 4, Treg in TexMACS formed small, loosely organized clusters, showing early signs of colony formation. However, cells were mostly dispersed with fewer clusters in RPMI medium (Supplementary Fig. 2). By day 7, larger and more compact colonies were observed in Treg cultured in TexMACS, indicating robust proliferation, while Treg in RPMI showed smaller, less defined clusters with fewer cells, indicating slower growth at day 7. The same pattern continued to day 14, where Treg displayed tightly packed and larger spheroids in TexMACS with almost confluent cells spread outside colonies compared to Treg cultured with RPMI, where empty spaces could be seen between cells spread outside colonies. By day 21, Treg in TexMACS maintained their spheroid colony shape with slightly reduced compactness, indicating a plateau in proliferation, while in RPMI, the clusters remained small, and loosely packed, reflecting limited growth. Overall, Treg exhibited slower growth in RPMI compared to TexMACS, indicating that RPMI was less conducive to Treg expansion (Supplementary Fig. 2).

**Fig. 1:**
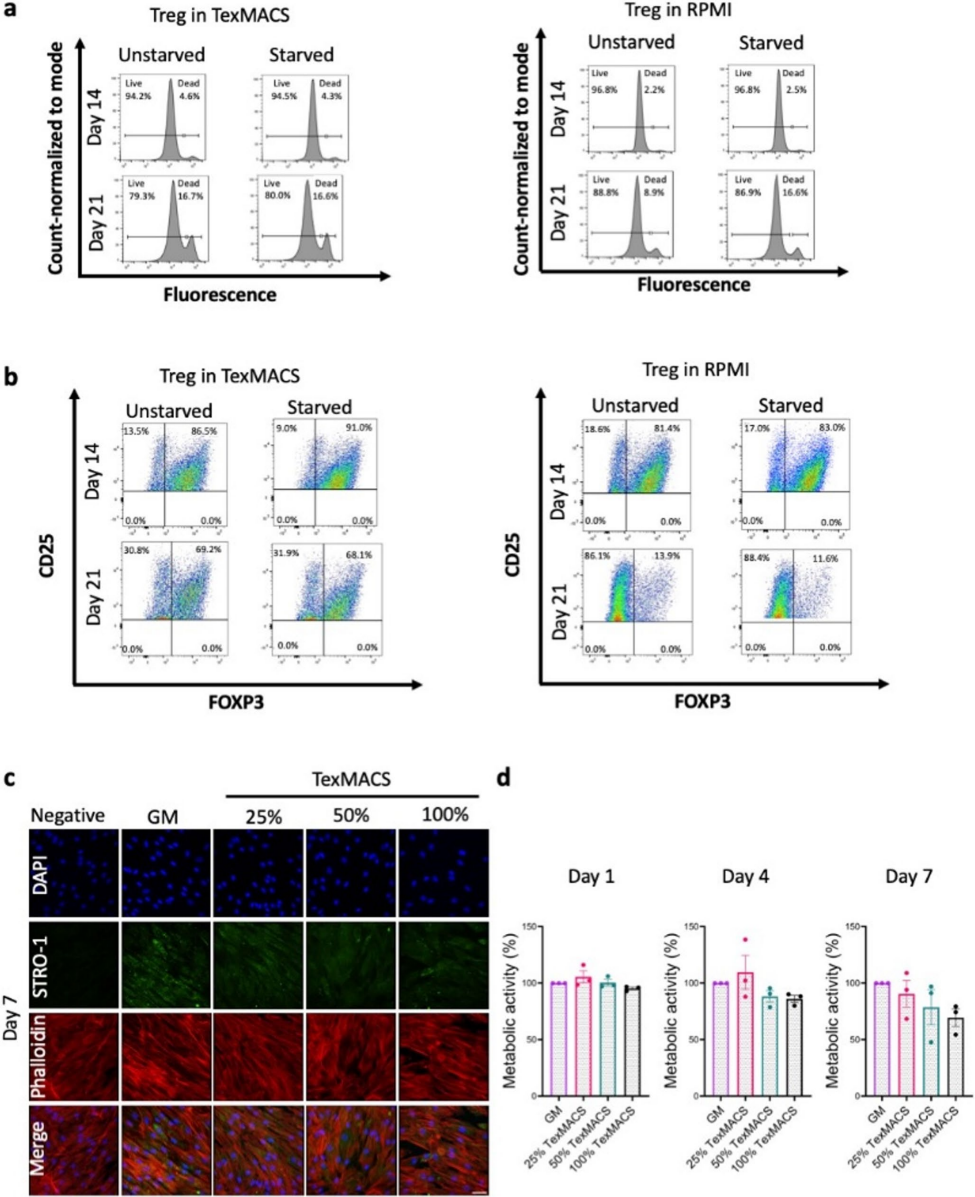
Optimization of culture conditions. **a** Viable and non-viable regulatory T cells (Treg) from one donor cultured in RPMI or TexMACS media, with and without starvation, at days 14 and 21. **b** Characterization of Treg cultured in TexMACS or RPMI with and without starvation, at days 14 and 21. **c** Phalloidin staining for F-actin (red), DAPI staining for nuclei (blue), and STRO-1 expression (green). Scale bar: 50 μm. **d** Metabolic activity of bone marrow mesenchymal stromal cells (BMSC) cultured in growth media (GM) or TexMACS. Data are presented as mean ± standard error of mean from three independent donors (*n* = 3) *p ≤ 0.05, **p ≤ 0.01, ***p ≤ 0.001, ****p ≤ 0.0001

When cultured in TexMACS, Treg cell death increased from 4.6% to 16.7% under non-starved conditions and from 4.3% to 16.6% with starvation. In RPMI, cell death increased from 2.2% to 8.9% in non-starved conditions and from 2.5% to 16.6% under starvation. Overall, the prolonged incubation led to a greater percentage of dead cells in both groups after 21 days compared to 14 days, regardless of starvation (Fig. 1a). Furthermore, the percentage of CD25^⁺^FOXP3^⁺^ Treg within theCD45^⁺^CD4^⁺^CD127^dim/−^ population decreased from day 14 to day 21. However, the decline was more pronounced in RPMI (from 81.4% to 13.9% unstarved and from 83.0% to 11.6% starved) compared to TexMACS (from 86.6% to 69.2% unstarved and from 91.0% to 68.1% starved). This confirms that TexMACS preserved Treg viability and phenotype (Fig. 1b).

BMSC were characterized as CD34^−^HLA-DR^−^CD45^−^ while expressing CD90^+^CD73^+^CD105^+^ (Supplementary Fig. 3a) and multipotency was confirmed by ostoeogenic and adipogenic differentiation (Supplementary Fig. 3b). Results showed that as the concentration of TexMACS increased, BMSC exhibited reduced cytoskeletal organization and cell density compared to GM after 7 days of culture. STRO-1 expression, also decreased with increasing TexMACS concentration, indicating a potential loss of stemness at higher concentrations (Fig. 1c). BMSC metabolic activity was slightly decreased in a dose-dependent manner in response to TexMACS, with 100% TexMACS maintaining the lowest metabolic activity (Fig. 1d). Although no significant differences were observed between cells cultured in GM and cells treated with TexMACS at any time point, these results suggested that TexMACS medium has a slightly negative effect on both BMSC morphology and metabolic activity and as such Non-CM (cell-free TexMACS) was included as a control.

### Treg-CM Modulates BMSC Metabolic Activity, Morphology and Migration Patterns

**Fig. 2:**
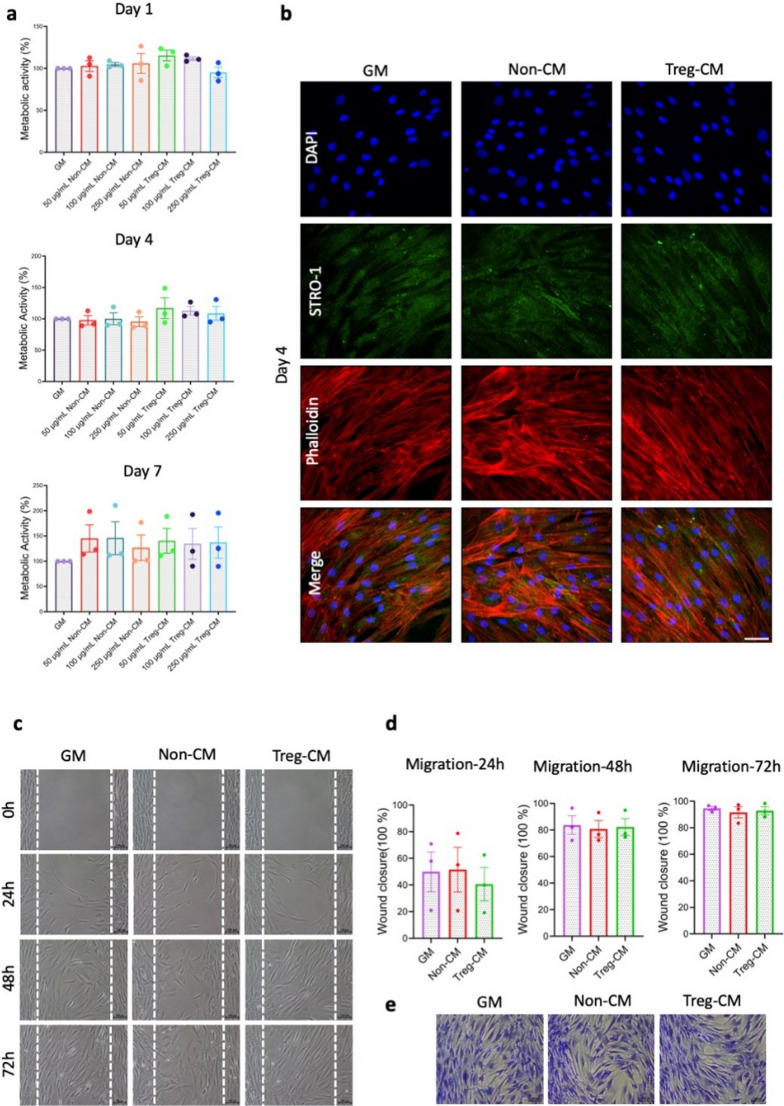
Effect of Treg condition medium (Treg-CM; 50 µg/mL) or non-condition medium (Non-CM; 50 µg/mL) on bone marrow mesenchymal stromal cells (BMSC) metabolic activity, morphology, and migration. **a** BMSC metabolic activity on days 1, 4, and 7. **b** BMSC treated with Treg-CM on day 4, with GM and Non-CM as controls. Phalloidin staining for F-actin (red), DAPI staining for nuclei (blue), and STRO-1 expression (green). Scale bar: 50 μm. **c** Migration of BMSC treated with Treg-CM. **d** Quantification of BMSC migration **e** Crystal violet staining of migrated BMSC treated with Treg-CM after 72 h, with GM and Non-CM as controls. Data are presented as mean ± standard error of mean from three independent donors (*n* = 3). *p ≤ 0.05, **p ≤ 0.01, ***p ≤ 0.001, ****p ≤ 0.0001

Initial experiments with percentage-based Treg-CM (25%, 50%, 100%) showed variable effects on BMSC metabolic activity at different time points. On day 4, BMSC metabolic activity was significantly higher with 25% Treg-CM compared to 25% Non-CM. On day 7, 50% Treg-CM significantly increased metabolic activity compared to 50% Non-CM (Supplementary Fig. 4a). We therefore next tested defined protein concentrations of Treg-CM (50, 100, and 250 µg/mL) to refine our initial findings. There were no statistically significant differences found between Treg-CM and Non-CM in any of the tested concentrations or time point. However, notably, BMSC showed a consistent trend towards higher metabolic activity with the lowest concentration, 50 µg/mL Treg-CM, across all time points when compared to the other concentrations. This concentration also showed a consistent increase in metabolic activity when compared to the same concentration of Non-CM in the early time points. (Fig. 2a). Therefore, 50 µg/mL Treg-CM was chosen as the optimal concentration for further experiments.

The expression of STRO-1 and phalloidin at day 4 were assessed by immunofluorescence staining after treatment with 50 µg/mL Treg-CM or Non-CM. Results showed that STRO-1 expression was detected in all groups and control, indicating the maintenance of stemness. Although all groups showed well-organized actin filaments, denser and more bundled actin filaments were observed in BMSC treated with Treg-CM (Fig. 2c).

Furthermore, BMSC maintained their migratory ability when treated with 50 µg/mL Treg-CM, whereby no statistically significant differences were observed between groups at any time point (Fig. 2c-d). After 72 h of migration, crystal violet stain revealed BMSC preserved their fibroblast-like morphology across all conditions (Fig. 2e).

### Treg-CM Modulates BMSC Osteogenesis at the Gene Level

**Fig. 3:**
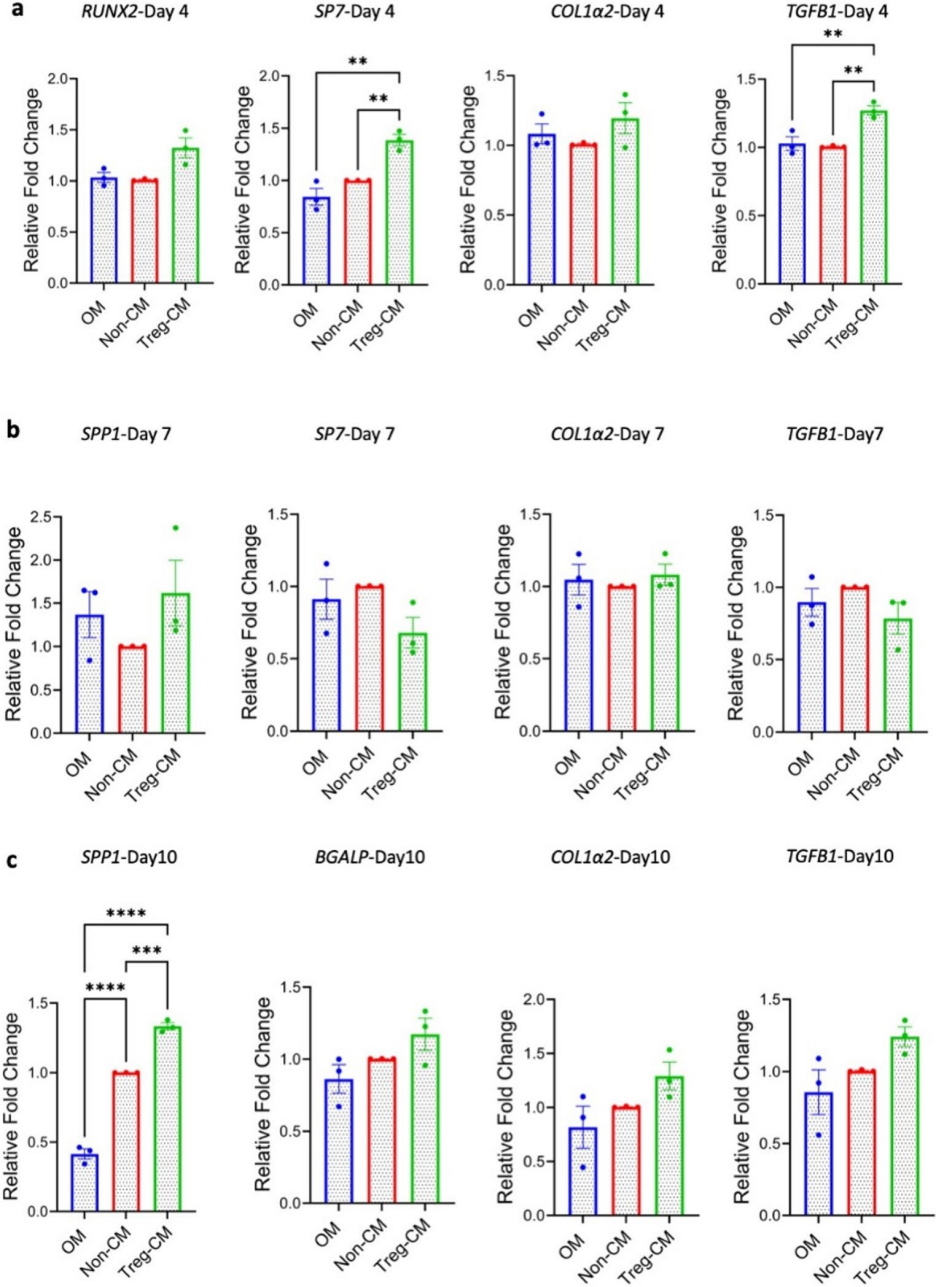
Gene expression of bone marrow mesenchymal stromal cells (BMSC) treated with Treg condition medium (Treg-CM; 50 µg/mL) or non-condition medium (Non-CM; 50 µg/mL) in osteogenic medium (OM). a Early osteogenic differentiation at day 4. **b** Early and late osteogenic differentiation on day 7. **c** Late osteogeneic differentiation on day 10. Data are presented as mean ± standard error of mean from three independent donors (*n* = 3). *p ≤ 0.05, **p ≤ 0.01, ***p ≤ 0.001, ****p ≤ 0.0001

The expressions of key osteogenesis-related genes were assessed in BMSC after culture with 50 µg/mL Treg-CM. On day 4, *SP7* and *TGFB1* were significantly upregulated in Treg-CM treated BMSC, while *RUNX2* and *COL1α2* expression showed an increase compared to BMSC in OM and Non-CM, albeit insignificant (Fig. 3a).

As differentiation progressed to day 7, an increase in the expression of the late osteogenic marker *SPP1* and a decrease in the early differentiation marker *SP7* were observed in the Treg-CM group compared to Non-CM and OM, however not significant (Fig. 3b).

After 10 days, *SPP1* expression was significantly higher in BMSC exposed to Treg-CM compared to both Non-CM and OM. Additionally, *BGLAP* expression showed a non-significant upregulation in Treg-CM-treated BMSC compared to OM. Furthermore, *COL1α2* and *TGFB1* expressions were also increased on day 10 in the Treg-CM group compared to OM, albeit statistically non-significant (Fig. 3c).

### Treg-CM Promotes BMSC’s Matrix Deposition and Enhanced Mineralization

To initially explore the effects of Treg-CM on BMSC osteogenic differentiation, BMSC were treated with Treg-CM (10%, 25% or 50%). Treg-CM in 25% significantly increased ALP activity on day 4 compared to 25% Non-CM, while 50% Treg-CM significantly elevated ALP activity on day 7 compared to 50% Non-CM (Supplementary Fig. 4b). Further assessments at day 14 showed that ALP staining and mineralization were significantly higher in the 25% Treg-CM group compared to 25% Non-CM (Supplementary Fig. 4c-d-e), demonstrating a dose-dependent impact on BMSC osteogenic differentiation.

**Fig. 4:**
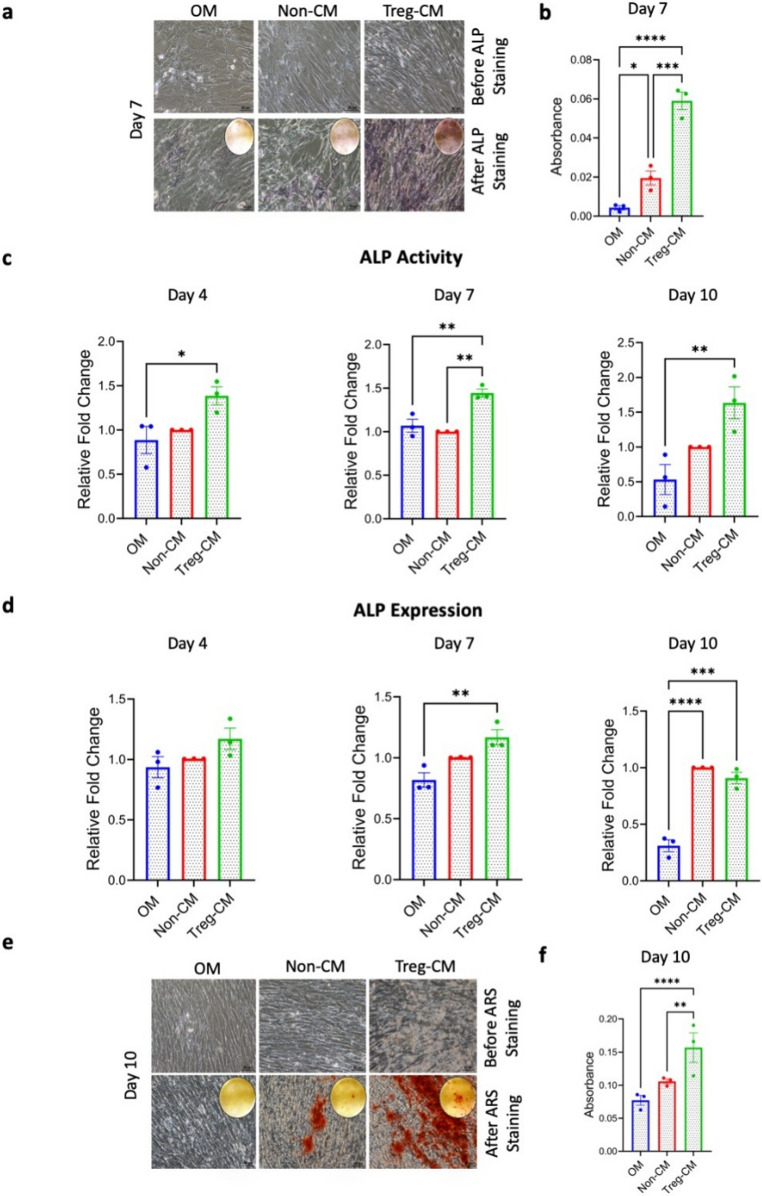
Osteogenic potential of bone marrow mesenchymal stromal cells (BMSC) treated with Treg condition medium (Treg-CM; 50 µg/mL) or non-condition medium (Non-CM; 50 µg/mL) in osteogenic medium (OM). **a** Alkaline phosphatase* (*ALP) staining on day 7. Scale bar: 50 μm. **b** Quantification of ALP staining. **c** ALP enzymatic activity normalized to cell number and expressed as a fold change relative to cells cultured in Non-CM **d** *ALP* gene expression. **e** Alizarin red S (ARS) staining on day 10. **f** Quantification of ARS staining*.* Scale bar: 50 μm. Data are presented as mean ± standard error of mean from three independent donors (*n* = 3). *p ≤ 0.05, **p ≤ 0.01, ***p ≤ 0.001, ****p ≤ 0.0001

To evaluate further the effect of Treg-CM on BMSC osteogenic differentiation, ALP staining was performed at day 7 after BMSC were culture with pre-defined concentration of Treg-CM (50 µg/mL). ALP staining was significantly increased on day 7 in BMSC treated with 50 µg/mL Treg-CM compared to OM and Non-CM (Fig. 4a-b). This observation was further supported by ALP enzyme activity significantly increased on days 4, 7, and 10 in BMSC treated with Treg-CM compared to OM. Notably, ALP activity on day 7 was significantly higher in BMSC treated with Treg-CM compared to both Non-CM and OM (Fig. 4c). At mRNA level, *ALP* gene expression was also significantly upregulated on day 7 and 10 in the Treg-CM group compared to OM (Fig. 4d). By day 10, BMSC treated with Treg-CM exhibited significantly higher calcium deposition compared to controls, confirming an accelerated matrix mineralization (Fig. 4e-f).

### Treg-CM Proteomics Profile Shows Enrichment in Immune Modulation and Cytoskeletal Regulation

**Fig. 5:**
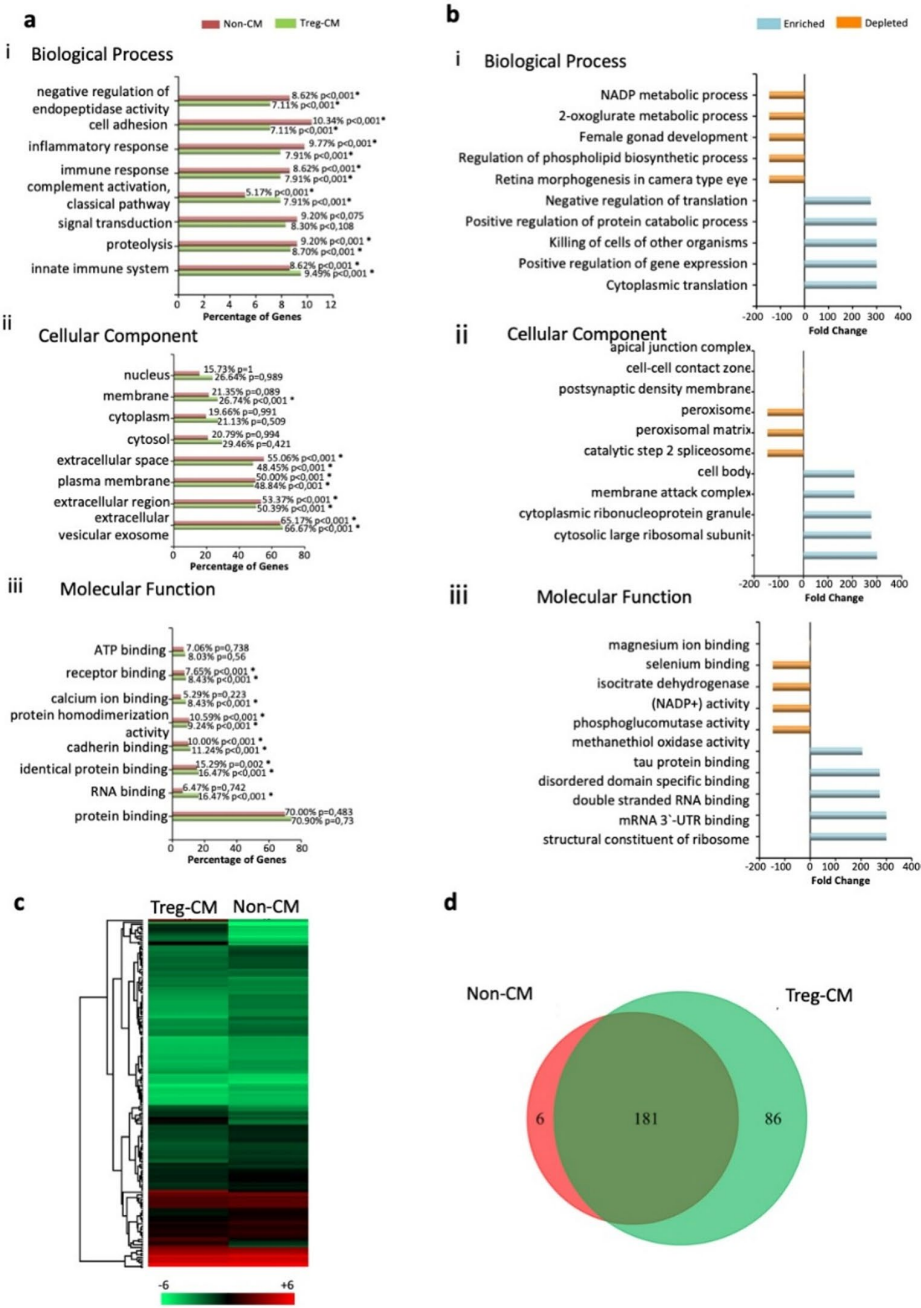
Functional enrichment analysis of total proteins in Treg condition medium (Treg-CM) and non-condition medium (Non-CM). **a** Bar diagrams representing the top 8 enriched terms in **(i)** biological processes, **(ii)** cellular components, and **(iii)** molecular functions. Significance against the gene ontology* (*GO) dataset is marked by an asterisk. **b** GO-based analysis of **(i)** biological processes, **(ii)** cellular components, and **(iii)** molecular functions enriched or depleted in proteins differentially abundant in Treg-CM compared to Non-CM. **c** Heat map of hierarchically clustered, protein expression levels between Treg-CM and Non-CM, based on log₂-transformed expression values. **d** Venn diagram showing the number of unique and shared genes between Non-CM (red) and Treg-CM (green)

Functional enrichment analysis of total proteins in Treg-CM and Non-CM against GO databases revealed that Treg-CM exhibited a higher percentage of proteins associated with the innate immune system (*p* < 0.001) and complement activation classical pathway (*p* < 0.001) compared to Non-CM (Fig. 5ai). Treg-CM was also significantly enriched in extracellular vesicular exosomes (*p* < 0.001) and membranes (*p* < 0.01) (Fig. 5aii). Regarding molecular functions, Treg-CM showed a higher percentage of proteins involved in RNA binding (*p* < 0.001), cadherin binding (*p* < 0.001), calcium ion binding (*p* < 0.001), and receptor binding (*p* < 0.001) (Fig. 5aiii). The distinct differences identified in the initial enrichment analysis led to a more focused fold-change enrichment analysis to explore the specific proteins that are differentially expressed and enriched on Treg-CM.

Treg-CM is notably enriched in proteins involved in key biological processes, including cytoplasmic translation and the positive regulation of gene expression, reflecting its role in protein synthesis and transcriptional activation (Fig. 5bi). Additionally, Treg-CM exhibited higher levels of proteins associated with the cytosolic large ribosomal subunit and cytoplasmic ribonucleoprotein granules, essential for mRNA translation and stability (Fig. 5bii). Molecular function analysis revealed that proteins predominantly enriched in Treg-CM serve as structural constituents of ribosomes and bind to the 3’ untranslated region (UTR) of mRNA, crucial for maintaining ribosomal structure and regulating mRNA stability and translation (Fig. 5biii).

Distinct protein expression levels between Treg-CM and Non-CM were visualized in a heat map **(**Fig. 5c**)** indicating a wide range of protein expression levels in both Treg-CM and Non-CM. Venn diagram showed a total of 181 proteins shared between Treg-CM and Non-CM, while 86 proteins were unique to Treg-CM (Fig. 5d). The top 30 most abundant proteins unique to Treg-CM are listed in Table 1, and a full list of unique proteins are in Supplementary Table S5.

**Table 1:** The top 30 most abundant proteins uniquely identified in Treg-CM and their functions based on UniProt. Data were retrieved from the UniProt database (UniProt, 2025)

Gene Symbol	Protein Name	Abundances	Function
H2BC12	Histone H2B type 1-K	24.6814	A core component of the nucleosome, crucial for chromosomal stability, DNA packaging, and gene regulation.
CFH	Complement factor H	24.5904	A glycoprotein that prevents complement activation and amplification on cell surfaces.
PLG	Plasminogen	24.5664	A zymogen of plasmin involved fibrin degradation, tissue remodeling, and tumor invasion.
C4A	Complement C4-A	24.4245	Essential for the propagation of the classical complement pathway.
GAPDH	Glyceraldehyde-3-phosphate dehydrogenase	24.4179	Involved in glycolysis and regulates cytoskeletal and nuclear events, as well as immune responses.
ITIH2	Inter-alpha-trypsin inhibitor heavy chain H2	24.1979	Similar to ITIH1, modulating the localization, synthesis, and degradation of hyaluronan.
ITIH1	Inter-alpha-trypsin inhibitor heavy chain H1	24.0208	Modulates the localization, synthesis, and degradation of hyaluronan.
APOB	Apolipoprotein B-100	23.9932	Acts as a recognition signal for the cellular binding and internalization of low-density lipoprotein (LDL).
MSN	Moesin	23.8642	Connects the actin cytoskeleton to the plasma membrane, regulating cell structure and immunity.
RNF10	E3 ubiquitin-protein ligase RNF10	23.6036	Catalyzes the monoubiquitylation of 40 S ribosomal proteins.
IGHG4	Immunoglobulin heavy constant gamma 4	23.3712	Represents the constant region of immunoglobulin heavy chains.
C4BPA	C4b-binding protein alpha chain	23.0238	Controls the classical pathway of complement activation.
TRAPPC10	Trafficking protein particle complex subunit 10	22.6835	A subunit of the transport protein particle II, involved in Golgi trafficking.
ACTC1	Actin, alpha cardiac muscle 1	22.4203	A key structural protein involved in cell motility.
HSP90AB1	Heat shock protein HSP 90-beta	22.2375	A chaperone protein involved in protein maturation, stabilization, transcription, and immune response.
HSP90AA1	Heat shock protein HSP 90-alpha	22.1785	A chaperone protein involved in protein maturation, stabilization, transcription, and immune response.
F2	Prothrombin	21.9848	A precursor to thrombin involved blood homeostasis, inflammation, and wound healing.
IGHV4-39	Immunoglobulin heavy variable 4–39	21.9214	V region of the variable domain of immunoglobulin heavy chains, crucial for antigen recognition.
WDR1	WD repeat-containing protein 1	21.7972	Modulates actin dynamics, cytokinesis, cell migration, and epithelial organization.
IGHV3-7	Immunoglobulin heavy variable 3–7	21.7509	V region of the variable domain of immunoglobulin heavy chains, crucial for antigen recognition.
CORO1A	Coronin-1 A	20.9838	A component of the cytoskeleton of highly motile cells.
PKM	Pyruvate kinase PKM	20.908	Involved in glycolysis.
C9	Complement component C9	20.8804	A component of the membrane attack complex, involved in the immune response.
LDHA	L-lactate dehydrogenase A chain	20.657	Catalyzes the conversion of pyruvate to lactate and vice versa, important for cellular metabolism.
HLA-A	HLA class I histocompatibility antigen, A alpha chain	20.471	An antigen-presenting major histocompatibility complex class I (MHCI) molecule.
ARHGDIB	Rho GDP-dissociation inhibitor 2	20.4114	Regulates GDP/GTP exchange in Rho proteins and the reorganization of the actin cytoskeleton.
C7	Complement component C7	20.3686	A component of the membrane attack complex, involved in both innate and adaptive immune responses.
TUBA1B	Tubulin alpha-1B chain	20.3036	A major component of microtubules.
PROS1	Vitamin K-dependent protein S	19.8837	An anticoagulant plasma protein.
C1S	Complement C1s subcomponent	19.8727	A serine protease involved in the complement system.

**Fig. 6:**
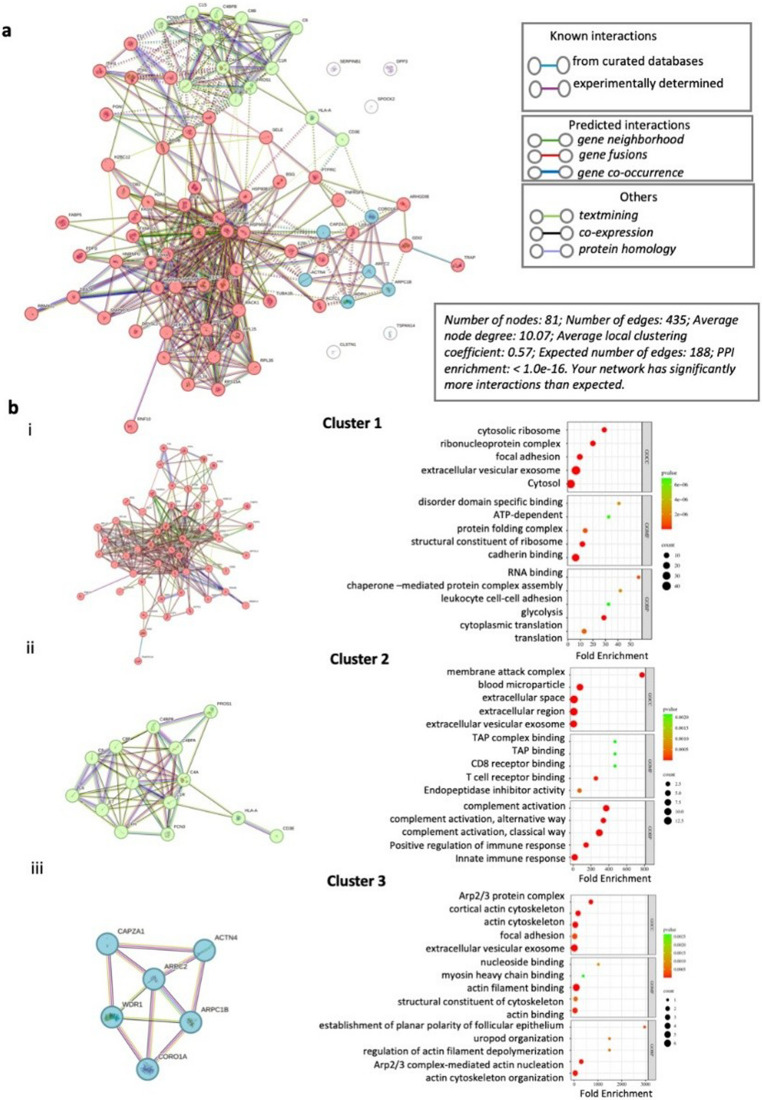
Protein-protein interaction network and functional enrichment analysis of differentially expressed unique proteins in regulatory T cell-condition medium (Treg-CM). **a** The network generated by STRING. Nodes represent proteins, and edges represent protein-protein associations. **b** Cluster analysis with k = 3 identified three main clusters in Treg-CM and shown as **(i)** cluster 1, **(ii)** cluster 2 and **(iii)** cluster 3 using STRING. Each cluster further analyzed for functional enrichment by FunRich using gene ontology (GO) databases. The top five enriched terms for cellular compartment, and molecular function and biological processes are visualized using SR Plot. The size of the dots indicates the number of proteins associated with each term, while the color gradient represents the p-value of the enrichment analysis

A deeper analysis on the 86 unique proteins in Treg-CM identified a complex network of interactions with significant enrichment score (p-value < 1.0e-16) (Fig. 6a). The PPI network in Cluster 1 showed significant score (p-value < 1.0e-16) in biological processes such as “cytoplasmic translation” and “glycolysis,” indicating roles in protein synthesis and energy metabolism. This cluster was associated with cellular components like the “cytosol” and “extracellular vesicular exosome,” essential for intracellular transport and extracellular communication, respectively. Molecular function analysis revealed enrichment in “RNA binding” and “cadherin binding,” which are critical for mRNA regulation and cell-cell adhesion (Fig. 6bi). The PPI network in Cluster 2 showed significant enrichment score (p-value < 1.0e-16) in biological processes in “immune response”. Key cellular components included the “extracellular exosome” and “membrane attack complex,” indicating their roles in extracellular communication and immune defense. Molecular function analysis showed “receptor binding”, indicating this cluster’s role in signal transduction and cellular response to external stimuli (Fig. 6bii). The PPI network in Cluster 3 showed significant enrichment score (p-value < 1.55e-15) in “actin cytoskeleton organization” and “uropod organization,” which are crucial for maintaining cell morphology, motility, and cellular interactions. This cluster was associated with cellular components like the “extracellular vesicular exosome” and “focal adhesion”. Enriched molecular functions included “actin binding,” indicating a role in cytoskeletal organization and cell structure maintenance (Fig. 6biii).

### Distinct Osteogenic Cytokines in Treg-CM and BMSC

**Fig. 7:**
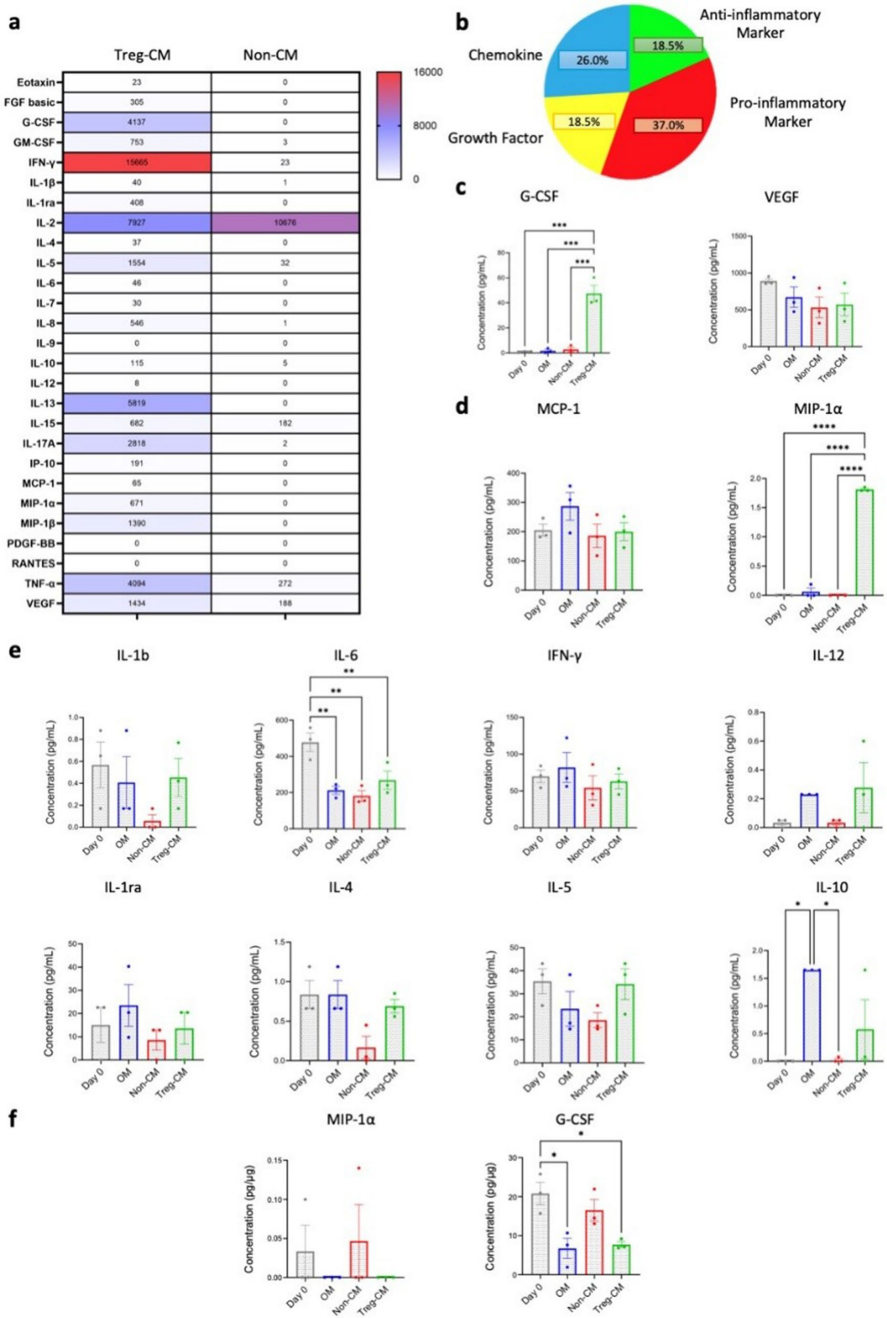
Cytokine analysis in Treg condition medium (Treg-CM) and non-condition medium (Non-CM) or bone marrow mesenchymal stromal cells (BMSC) treated with Treg-CM (50 µg/mL) or Non-CM (50 µg/mL) in osteogenic medium (OM. **a** Heatmap shows cytokine concentrations (pg/mL) detected in Treg-CM and Non-CM. **b** Pie chart showing the percentages of growth factors, chemokines and pro-inflammatory cytokines, anti-inflammatory cytokines detected in Treg-CM **c** Growth factor levels in the culture supernatant of BMSC at day 7. **d** Chemokine levels in the culture supernatant BMSC at day 7. **e** Pro-inflammatory/anti-inflammatory cytokine levels in supernatant of BMSC at day 7. **f** MIP-1α and G-CSF levels in BMSC lysate at day 7. Data are presented as mean ± standard error of mean from three independent donors. *p ≤ 0.05, **p ≤ 0.01, ***p ≤ 0.001, ****p ≤ 0.0001

The multiplex immunoassay portrayed increased levels of multiple cytokines, including interleukins (e.g., IL-10, IL-13), interferons (e.g., IFN-γ), growth factors (e.g., VEGF, GM-CSF), and chemokines (e.g., MIP-1α, MIP-1β, IP-10) in Treg-CM compared to Non-CM, while only IL-2 was higher in Non-CM compared to Treg-CM. Its high levels in Non-CM are likely due to absence of Treg, however IL-2 was excluded from the analysis as it was supplemented in the culture media (Fig. 7a). Cytokines in Treg-CM were categorized as 26% chemokines, 18.5% growth factors 37% pro-inflammatory cytokines, 18.5% anti-inflammatory cytokines (Fig. 7b).

G-CSF levels in BMSC supernatant were significantly increased in Treg-CM-treated BMSC compared to OM, Non-CM, and BMSC alone at day 0, while VEGF levels in the supernatant were slightly lower in Treg-CM-treated BMSC compared to day 0 **(**Fig. 7c**)**. MCP-1 levels in the supernatant decreased in Treg-CM compared to OM, while MIP-1α levels were significantly higher in Treg-CM-treated BMSC supernatants compared to OM, Non-CM, and day 0 (Fig. 7d). IL-6 levels in the supernatant were significantly decreased in BMSC treated with Treg-CM, OM, and Non-CM compared to day 0. IL-1β, IFN-γ, and IL-6 levels in the supernatant showed higher trends in Treg-CM-treated BMSC compared to Non-CM, albeit not statistically significant. IL-1ra, IL-4, and IL-5 levels in the supernatant were slightly higher in Treg-CM-treated BMSC compared to Non-CM, though insignificant. IL-10 levels in the supernatant were significantly higher in OM compared to BMSC at day 0 and Non-CM. Additionally, IL-10 levels in the supernatant showed a slight but insignificant increase in BMSC treated with Treg-CM compared to treated with Non-CM and BMSC alone at day 0, comparable to OM (Fig. 7e). The absolute concentrations of all cytokines from the supernatant are summarized in Supplementary Table S4. With respect to BMSC lysates, MIP-1α levels showed lower trends in the Treg-CM group compared to BMSC alone at day 0 and Non-CM, while G-CSF levels were significantly reduced in BMSC treated with OM and Treg-CM compared to BMSC alone at day 0 **(**Fig. 7f**).**

Additionally, correlation analysis between cytokine levels and osteogenic outcomes in BMSC treated with Treg-CM revealed most of the cytokines such as VEGF-A, IFN-γ, MCP-1, MIP-1α, and G-CSF were positively correlated with each other and mineralization. Notably, ALP staining showed weaker correlation with other cytokines compared to mineralization. However, these correlations did not reach statistical significance (Supplementary Fig. S5).

## Discussion

The immune system is crucial for maintaining bone homeostasis [29]; our study provides new insights into how Treg indirectly modulates, through their secretome, the osteogenic potential of BMSC.

Treg are highly metabolic in culture, engaging both glycolysis and fatty-acid oxidation to sustain their proliferation and suppressive functions [30]. This dual metabolic strategy affects the composition of their secretome, which contains bioactive molecules that modulate the behavior of target cells. Several studies have highlighted that aerobic glycolysis is the predominant source of energy promoting the differentiation of BMSC [31–33]. Inhibition of glycolysis has been shown to suppress osteogenesis in primary bone marrow mesenchymal progenitors [31], while downregulation of glycolytic pathways in BMSC-like ST2 cells also leads to decreased osteogenesis and mineralization [32]. Our proteomics identified pyruvate kinase, phosphoglycerate kinase 1, glyceraldehyde-3-phosphate dehydrogenase in Treg-CM, which are key glycolytic enzymes, known to play essential roles in cellular energy metabolism and osteogenesis [34]. The presence of these enzymes suggests that Treg-CM may influence BMSC differentiation though metabolic signaling pathways. Although the overall metabolic activity of BMSC exposed to concentrated, standardized Treg-CM did not show statistically significant changes, we observed a consistent trend toward enhanced osteogenic differentiation. This implies that Treg-CM may induce a subtle but functionally relevant metabolic reprogramming, potentially favoring glycolytic flux, without altering total metabolic output. These findings warrant further investigation to delineate the specific metabolic shifts underlying Treg-CM mediated osteogenesis.

Furthermore, our proteomic profiling of Treg-CM showed the presence of proteins related to the actin cytoskeleton, including actin, alpha-actin, and the actin-related protein 2/3 complex, Rho GDP-dissociation inhibitor 2. Moreover, denser and more bundled actin filaments were observed in BMSC treated with Treg-CM. This suggests that Treg-CM may initially impact cytoskeletal organization by modulating actin filament dynamics and microtubule organization. Moreover, the unique proteins in Treg-CM revealed by proteomics, included moesin and ezrin from the ERM (ezrin, radixin, moesin) family, which mediate membrane-cytoskeleton adhesion, cell elasticity, and actin cytoskeleton organization, and potential targets for modulating cellular mechanics. These proteins serve as cross-linkers between the plasma membrane and actin filaments in various mesoderm-derived cell types [35, 36]. During osteogenic differentiation, the reorganization of actin filaments in human MSC provides more binding sites for ERM linkers, increasing membrane-cytoskeleton interaction and supporting their transition to osteoblast [37]. In line with this, our proteomic analysis of Treg-CM revealed several actin-regulating proteins. Additionally, early mineral deposition was observed in BMSC treated with Treg-CM at day 10. These results suggest Treg-CM can promote osteogenic differentiation through cytoskeletal regulation.

Differentiation of BMSC into pre-osteoblasts and osteoblasts is an intricate process regulated by the coordinated expression of specific genes [38]. Our proteomics revealed unique proteins in Treg-CM involved in pre-mRNA packaging, mRNA transport, splicing, and translation, including nuceleolin, heterogenous nuclear ribonucleoproteins, transformer-2 protein homolog beta. These findings suggest that Treg-CM modulates gene expression, leading to the enhancement of osteoblast-specific genes expressions. Indeed, BMSC treated with Treg-CM showed significant upregulation of early differentiation marker (*SP7*), followed by the upregulation of late-stage marker (*SPP1*) compared to those treated with Non-CM and OM; highlighting the impact of Treg-CM on both early and late stages of osteogenesis. I.-H. Kang et al. have previously shown that Treg-CM increases *RUNX2* expression during the early stages of osteogenic induction, albeit in mice osteoblasts. In the same study, *BGALP* (osteocalcin) expression, a key late differentiation marker, remained consistently higher in osteoblasts derived from wild type and osteogenesis imperfecta mice treated with Treg-CM throughout the culture period [7]. *RUNX2* expression occurs at early stages of osteochondroprogenitor determination, followed by *SP7* induction during osteoblast maturation [39]. Additionally, *SPP1* expression peaks twice during osteogenic differentiation: initially around day 4 during the proliferation stage and later between days 14 and 21 during the mineralization phase [40]. In our results, *SPP1* expression was significantly increased at day 10 in the Treg-CM-treated group, suggesting that Treg-CM modulates *SPP1* expression, potentially enhancing its role in promoting osteogenic differentiation and facilitating an earlier transition to mineralization. Additionally, *BGALP* expression, was higher in BMSC treated with Treg-CM compared to cells in OM, further supporting the role of Treg-CM in promoting late-stage osteogenic differentiation. This increase in *BGALP* expression aligns with enhanced mineralization, showing that Treg-CM facilitates not only osteogenic gene expression, but also functional matrix mineralization. To our knowledge, this is the first study to examine the effects of conditioned medium from expanded natural human Treg on human BMSC osteogenesis at a gene level. While the only comparable published study used committed mouse osteoblasts [7], and our results align with their results, highlighting Treg-CM’s potential to enhance and sustain osteogenic differentiation by regulating key osteoblast genes and modulating osteogenesis.

Another interesting bone related marker is TGF-β. In our study, *TGFB1* gene expression was significantly increased in BMSC treated with Treg-CM on day 4, indicating its potential role in promoting early osteoblast differentiation. Although *TGFB1* expression was not significantly changed at later time points, its expression pattern suggests dynamic, stage-dependent regulation during osteogenic differentiation. It has been shown that TGF-β signaling promotes osteogenic differentiation at early stages, and matrix maturation, but regulates matrix mineralization at later stages [41]. TGF-β in the Treg secretome is also suggested to activate MAPK and Smad signaling pathways, promoting MSC differentiation into osteoblasts [42]. In line with this, our pathway analysis of Treg-CM revealed enrichment of TGF-β receptor signaling, SMAD2/3 signaling, and p38-MAPK signaling pathways though it did not reach statistical significance (data not shown). Nonetheless, differences in experimental designs, including variations in time points, culture conditions, and stimuli used across studies, can lead to inconsistencies in findings, complicating direct comparisons and interpretations. Overall, our results suggest Treg-CM influences BMSC osteogenesis by modulating *TGFB1* expression in a stage-dependent manner, potentially facilitating early differentiation.

Further extending these findings, our results revealed that Treg-CM significantly increased ALP activity at day 7. ALP expressed by osteoblasts is crucial for bone formation; by hydrolyzing inorganic pyrophosphate, it increases inorganic phosphate concentration, promoting the formation of calcium phosphate or hydroxyapatite crystals in matrix vesicles. These crystals are essential for the initial stage of extracellular matrix mineralization [43]. Previous studies have shown that CD4^+^ T cells (directly), along with their secretome (indirectly), enhanced ALP activity in BMSC [20]. However, in the same study, 10% Treg-CM exhibited only a slight increase in ALP activity in BMSC, while higher concentrations appeared to have an opposite effect. In contrast, our study demonstrated that Treg-CM significantly enhanced ALP activity in BMSC. Unlike Croes et al., where naïve CD4^+^ T cells were differentiated into Treg, we directly isolated natural Treg from peripheral blood, which ensures a more functionally stable and immunosuppressive Treg population. Studies indicate that natural Treg show more stability and sustained suppressive function, driven by a distinct epigenetic profile, whereas induced Treg are more susceptible to losing suppressive function over time [44, 45]. Moreover, our optimized Treg-CM dosing and multiple time points assessment during osteogenesis allowed us to monitor dynamic changes in ALP activity that were not observed under the conditions used by Croes et al. Further, a recent study showed that adoptive transfer of Treg significantly improves bone, muscle, and skin tissue healing in mice, whereas delivery of CD4^+^ conventional T cells failed to promote healing [16]. These findings highlighted the unique ability of Treg and their secretome, particularly through IL-10, to positively modulate the bone regeneration compared to naïve CD4^+^ cells.

 Cytokine-producing Treg in circulation show significant heterogeneity, primarily due to their plasticity and ability to adapt to specific immune responses [46]. They can secrete Th1-related cytokines such as IFN-γ or Th2-related cytokines such as IL-4 and IL-13, while maintaining their immune homeostasis functions [46–48]. In fact, our cytokine analysis revealed distinct cytokine profiles present in Treg-CM while absent or less in Non-CM; including anti- or pro-inflammatory markers (e.g., IL-10, IL-4, IL-13, IFN-γ and TNF-α), growth factors (e.g., FGF-basic and GM-CSF), and chemokines (e.g., MIP-1α), which all have been shown previously to influence bone regeneration [16, 49–51]. Therefore, distinct cytokine profile of Treg-CM highlights the osteo-immunomodulatory profile of Treg with mixture of cytokines, likely acting synergistically to enhance BMSC osteogenesis. FGF-basic is a well-known mitogenic factor, and plays a major role in bone regeneration by promoting osteoblast proliferation, matrix formation, and mineralization [52]. Anti-inflammatory cytokines such as IL-4 and IL-13 can promote bone healing by modulating pro-healing macrophage response when delivered locally or enhance osteogenesis by increased ALP activity, collagen secretion and mineralization in vitro [53, 54]. In contrast, pro-inflamatory cytokines such as IFN-γ *and TNF-α* appears to exert a complex, likely context-dependent, both inhibitory and stimulatory role in osteogenesis [55–57]. Notably, priming MSC with IFN-γ and TNF-α induces secretion of paracrine mediators such as IL-6, HGF, VEGF, and TGF-β, which in turn enhance osteoblast proliferation and mineralization [58]. Since TGF-β is also secreted by Treg [42], and has been shown to block IFN-γ signaling [59], its presence in Treg-CM or BMSC cytokine mileu may likely counteract the inhibitory effect of IFN-γ and support osteoegenesis. Thus, complex paracrine signaling within Treg-CM likely act in synergy to prime BMSC to promote pro-osteogenic responseIt can also be postulated that the Treg-CM act in synergy with the osteogenic supplements in the culture to potentiate differentiation [60].

Interestingly, IL-6 levels were significantly decreased during osteogenic differentiation of BMSC treated with Treg-CM compared to baseline levels, consistent with its reported role in maintaining BMSC in a proliferative and undifferentiated state [61]. Since IL-6 shows similar dynamics among the BMSC treated with Treg-CM, Non-CM and OM, it may not be the primary driver of osteogenesis by the Treg secretome, while only affecting osteogenic differentiation in a context-dependent manner. Instead, we observed most of the cytokines, including VEGF-A, IFN-γ, MIP-1α, G-CSF, were positively correlated with each other and with mineralization in BMSC treated with Treg-CM. These findings suggest that these cytokines may collectively contribute to the osteo-inductive effects of Treg-CM. Although the correlations did not reach statistical significance, the observed significant increase in MIP-1α and G-CSF levels in the supernatant of BMSC treated with Treg-CM supports their potential involvement in osteogenic processes and suggests a biologically relevant trend that merits further investigation.

MIP1-α is a chemotactic chemokine and associated with both cell adhesion and migration and secreted by osteoblasts in active bone remodeling sites [62]. Since the BMSC were undergoing differentiation toward an osteogenic lineage, this could enhance the secretion of factors involved in bone remodeling. Additionally, MIP-1α has also been shown to modulate the adhesive phenotype of hematopoietic progenitors [63]. In our study, BMSC migration appeared slightly reduced after 24 h of Treg-CM treatment. This trend, along with increased MIP-1α levels in the BMSC supernatant, may indicate a shift toward an adhesive phenotype and enhanced attachment to the extracellular matrix.

Furthermore, G-CSF, a growth factor primarily secreted by fibroblasts, monocytes, macrophages, and T lymphocytes, has been shown to promote bone regeneration [50, 64]. In vivo, G-CSF facilities the mobilization of hematopoietic stem cells, MSC and endothelial progenitor cells by disrupting the CXCR4/SDF-1 axis [50]. However, its effects on MSC and osteoblasts appear to be context-dependent. Notably, G-CSF levels were significantly higher in BMSC supernatant following Treg-CM treatment in our study and G-CSF has been shown to upregulate β1 integrin in Swan 71 cells [65]. Our group and others have indeed previously shown that β1-integrins are key mediators of MSC adhesion, proliferation, as well as differentiation [66, 67]. This suggests a possible role for integrin signaling in the enhanced differentiation observed in Treg-CM-treated BMSC. Furthermore, locally applied G-CSF contributes to an ideal microenvironment for fracture healing by improving blood flow and promoting osteoblast recruitment to the defect [68, 69]. On the other hand, G-CSF alone has minimal effect on the proliferation and differentiation of human osteoblasts [70]. In the context of Treg-CM, the presence of G-CSF alongside with other cytokines may act synergistically to enhance osteogenesis in BMSC.

MIP-1α and G-CSF were both detected in Treg-CM, and their presence in the BMSC supernatant following Treg-CM treatment may result from either residual presence or active secretion by BMSC in response to the Treg secretome. Notably, cytokine levels were generally higher in the supernatant than in the cell lysates, and both MIP-1α and G-CSF were reduced in lysates from the Treg-CM and OM groups compared to Non-CM and day 0. This pattern suggests that these cytokines are secreted rather than stored intracellularly. Their release during osteogenic differentiation may contribute to driving a regenerative microenvironment that supports recruitment of immune cells and stem cells, tissue remodeling, and bone formation. Altogether, the presence of osteogenic-related cytokines on day 7, aligning with the upregulation of osteogenic gene expression, suggests that Treg-CM drives BMSC toward an osteoimmuno-modulatory state. Our findings, supported by the use of natural Treg, a standardized CM concentration and inclusion of Non-CM to confirm the observed *pro-osteogenic* effects were exclusively from Treg-CM, strengthen the validity of our approach. Additionally, despite using only three BMSC donors to assess the impact of Treg-CM, the consistent trends observed across all donors support the biological relevance of the findings and justify the choice based on feasibility and donor representativeness.

Nevertheless, our study presents with limitations, such as the contribution of individual components within Treg-CM and their molecular mechanisms are not fully stratified. Also, using pooled Treg-CM reduced donor variation but have hidden donor-specific effects. Furthermore, an in vitro study cannot capture the complexity of osteogenesis in vivo; therefore future studies should aim to identify these mechanisms and validate the potential of Treg-CM in vivo.

Taken together, our study indicates that Treg-CM dynamically regulates distinct phases of BMSC osteogenesis in vitro, from early differentiation to matrix mineralization. It upreguled the expression of key osteoblast-specific genes, and functionally increasing ALP activity and calcium deposition. Proteomic analysis identified that Treg-CM contains unique proteins involved in cytoskeletal organization, metabolic processes, and mRNA regulation. Additionally, cytokine profiling revealed osteoimmuno-modulatory potential of Treg-CM that may provide a pro-regenerative cues for BMSC. These insights highlight the synergistic effects of Treg-CM and its potential as a cell-free strategy to enhance BMSC osteogenesis.

## Supplementary Information

Below is the link to the electronic supplementary material.

ESM 1 (DOCX 3.58 MB)

## Data Availability

All data are provided in figures, tables and supplementary information or are otherwise available upon request.
