# Supplementary material for: Bone Marrow Mesenchymal Stromal Cell Osteogenesis is driven by Paracrine signals from Regulatory T Cell
Source: Stem Cell Rev Rep. 2025 Nov 10;22(1):636–56. doi: 10.1007/s12015-025-11015-2 (PMC12795956; doi:10.1007/s12015-025-11015-2)
Supplement: Supplementary file 1 — (DOCX 3.58 MB) [file 12015_2025_11015_MOESM1_ESM.docx]

**Bone Marrow Mesenchymal Stromal Cell Osteogenesis is Driven by Paracrine Signals from Regulatory T Cell**

Eylem Baysal^1^, Niyaz Al-Sharabi^1^, Kamal Mustafa^1^, Daniela E. Costea^2,3^, Meadhbh Brennan^4^, Salwa Suliman^1*^

1. Center of Translational Oral Research (TOR)—Tissue Engineering Group, Department of Clinical Dentistry, Faculty of Medicine, University of Bergen, Norway

2. The Gade Laboratory for Pathology and Center for Cancer Biomarkers (CCBIO) Department of Clinical Medicine, University of Bergen, Bergen, Norway.

3. Department of Pathology, Haukeland University Hospital, Bergen, Norway.

4. Regenerative Medicine Institute, School of Medicine, and Biomedical Engineering, School of Engineering, University of Galway, Galway, Ireland.

*Corresponding author:

Salwa Suliman, BDS, PhD, Associate Professor

Center for Translational Oral Research (TOR)

Department of Clinical Dentistry

Faculty of Medicine

University of Bergen

Årstadveien 19, 5009 Bergen, Norway

Tel: +47 55586356

Email: salwa.suliman@uib.no

**Supplementary Methods**

**1. Effect of Treg-CM at different percentages on BMSC metabolic activity**

BMSC (3 × 10^3^ cells/cm²) was seeded in GM and allowed to attach for 24 hours. Cells were treated with Treg-CM or Non-CM at 10%, 25% and 50%, diluted with GM (DMEM supplemented with 10% FBS and 1% p/s), for 7 days with media replaced twice a week. For each Treg-CM condition, the corresponding percentage of Non-CM (10%, 25%, or 50%) served as the control.

BMSC metabolic activity was assessed on day 4 and 7 using the PrestoBlue™ Cell Viability Reagent, following the manufacturer's protocol **(Section 2.2)**. Results were normalized to control, and cell viability expressed as a percentage relative to this control.

**2. Effect of Treg-CM at different percentages on BMSC ALP activity**

BMSC (3 × 10^3^ cells/cm²) was seeded in GM and allowed to attach for 24 hours. Cells were treated with Treg-CM or Non-CM at 10%, 25% and 50%, diluted with OM (DMEM, 10% FBS, 1% p/s supplemented with 173 µM L-ascorbic acid 2-phosphate, 10 nM dexamethasone, 10 mM β glycerophosphate), for 7 days with media replaced twice a week. For each Treg-CM condition, the corresponding percentage of Non-CM (10%, 25%, or 50%) served as the control.

BMSC ALP activity was assessed on day 4 and 7 using the Alkaline Phosphatase Yellow (pNPP) Liquid Substrate System for ELISA and the total cell number was quantified by using the PicoGreen™ assay, following the manufacturer's protocol. ALP activity was then normalized to cell number, and relative ALP activity was expressed as a percentage of the corresponding control.

BMSC were stained for ALP activity after 14 days. Cells were fixed with 4% paraformaldehyde, stained with BCIP®/NBT Liquid Substrate System (Sigma-Aldrich, St. Louis, MO, US) according to the manufacturer’s instructions. The stain was dissolved in cetylpyridinium chloride (Sigma-Aldrich, St. Louis, MO, US) overnight at room temperature for quantification using a microplate reader at 540 nm absorbance.

**3. Effect of Treg-CM at different percentages on BMSC mineralization**

BMSC (3 × 103 cells/cm²) was seeded in GM and allowed to attach for 24 hours. Cells were treated with Treg-CM or Non-CM at 10%, 25% and 50%, diluted with OM for 14 days with media replaced twice a week. For each Treg-CM condition, the corresponding percentage of Non-CM (10%, 25%, or 50%) served as the control.

BMSC were stained for calcium deposition and matrix mineralization after 14 days. Cells were fixed with 4% paraformaldehyde, stained with 2% Alizarin red S for 30 min. The stain was dissolved in cetylpyridinium chloride (Sigma-Aldrich, St. Louis, MO, US) 2 hours at room temperature and quantified using a microplate reader at 540 nm absorbance.

**Supplementary Results**


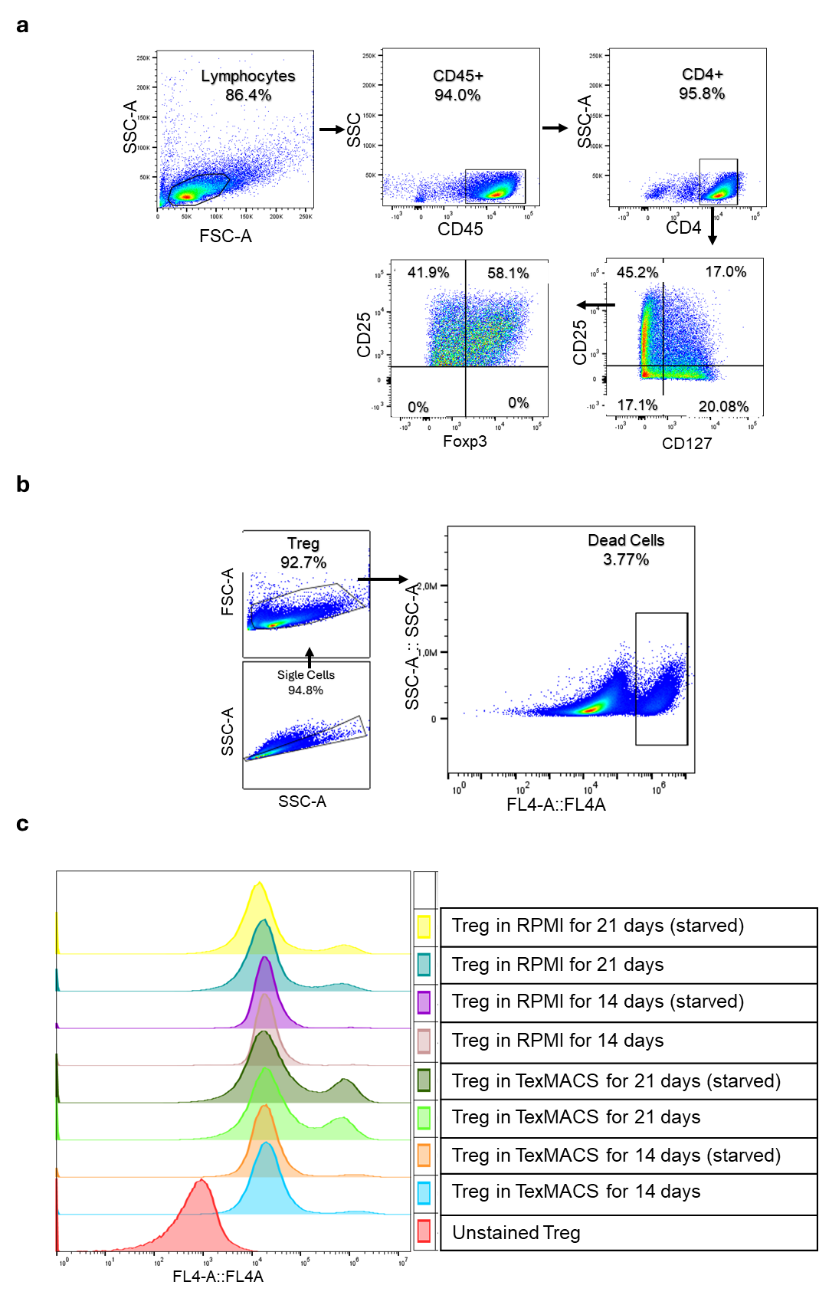


**Supplementary Fig. S1** **a** Treg flow cytometry characterization and gating **b** Treg Live/Dead assay gating **c** Comparison of Treg viability across RPMI or TexMACS culture conditions.

**
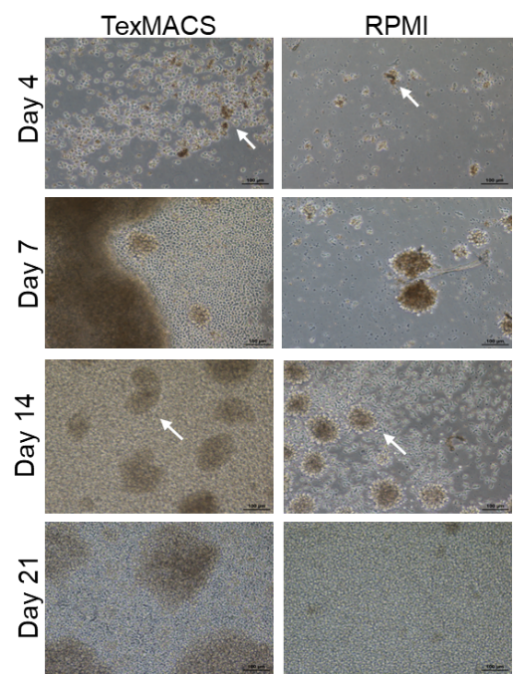
**

**Supplementary Fig. S2** Phase-contrast images of Treg in TexMACS or RPMI media over 21 days. Arrows in day 4 and day 14 panels indicate Treg clusters and cells spread outside the colonies, illustrating differences in morphology and density between media conditions. Scale bar: 100 µm.


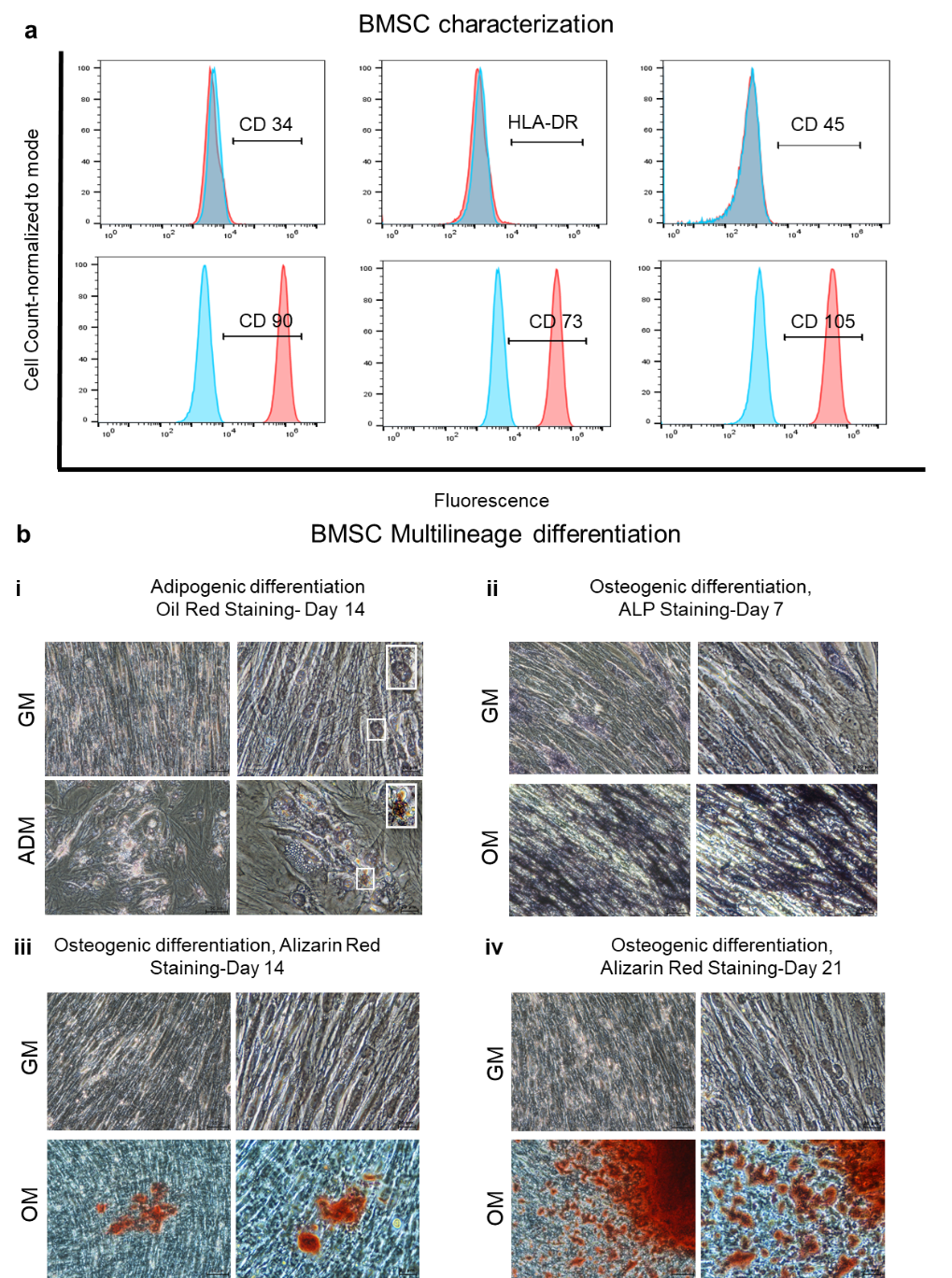


**Supplementary Fig. S3** **a** BMSC characterization and phenotyping by flow cytometry. **b** Adipogenic and osteogenic differentiation of BMSC.  **(i)** Oil Red O Staining of BMSC on day 14. **(ii)** Alkaline phosphatase staining of BMSC on day 7 **(iii)** Alizarin Red S staining of BMSC on days 14 and **(iv)** days 21. Magnifications 20× and 40×; Scale bars: 50 µm and 20 µm, respectively.


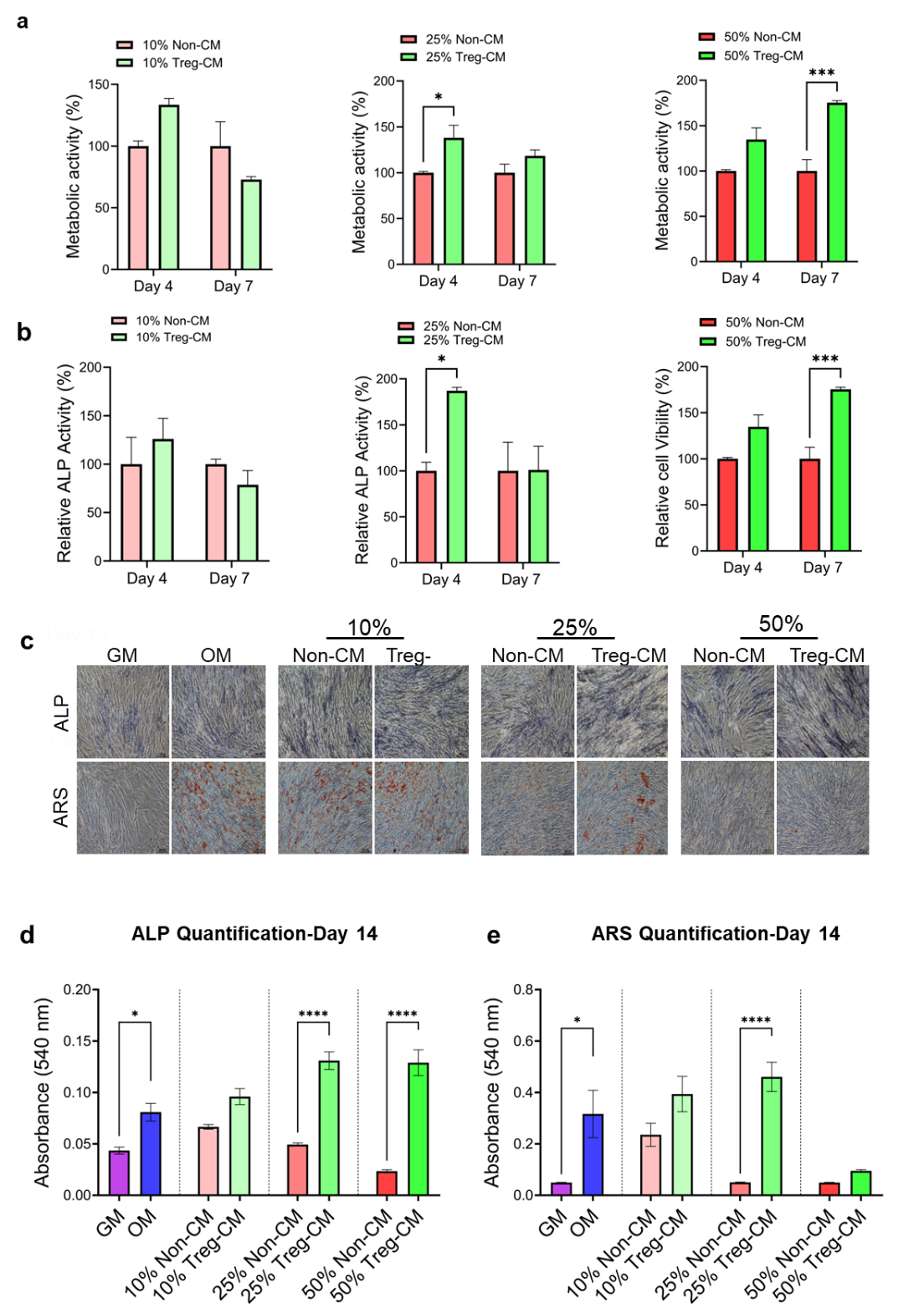


**Supplementary Fig. S4** **a** Metabolic activity of BMSC cultured in GM supplemented with 10%, 25%, and 50% Treg-condition medium (Treg-CM) compared to non-condition medium (Non-CM) controls (10%, 25%, and 50%). **b** Alkaline phosphatase activity of BMSC cultured in OM with Treg-CM compared to Non-CM. **c** ALP and Alizarin Red S stain on day 14 for BMSC cultured in OM with Treg-CM. Scale bar: 100 µm. **d** Quantification of ALP staining. **e** Quantification of Alizarin Red S staining. Data are presented as mean ± SEM (n = 1). *p < 0.05, **p < 0.01, ***p < 0.001.

**
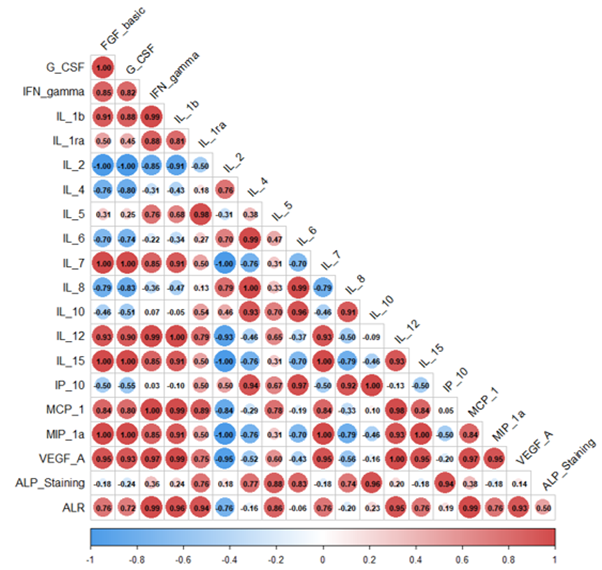
**

**Supplementary Figure S5:** Pearson’s Correlation coefficients matrix between detected cytokine levels in BMSC supernatant and osteogenic outcomes, alkaline phosphatase staining (ALP_Staining) and Alizarin red staining (ALR) in BMSC treated with 50 µg/mL Treg-CM. Red indicates positive correlations and blue indicates negative correlations, with circle size and intensity reflecting correlation strength.

**Supplementary Table S1.** Flow cytometry analysis of Treg under starved and non-starved culture conditions. N = 1

|  | **CD45^+^** | | **CD45+**  **CD4+** | | **CD45+**  **CD4+**  **CD127dim/-CD25+** | | **CD45+**  **CD4+**  **CD127dim/-**  **CD25+**  **FOXP3+** | |
| --- | --- | --- | --- | --- | --- | --- | --- | --- |
| **Starvation** | - | + | - | + | - | + | - | + |
| **TexMACS**  Day14 | 99.2 % | 99.1 % | 88.8 % | 90.0 % | 39.4 % | 37.7 % | 86.4 % | 91.0 % |
| **TexMACS**  Day21 | 98.6 % | 99.2 % | 45.3 % | 45.6 % | 37.9 % | 36.8 % | 69.2 % | 68.1 % |
| **RPMI**  Day14 | 99.3 % | 99.3 % | 89.8 % | 90.5 % | 42.7 % | 43.1% | 81.4 % | 83.0 % |
| **RPMI**  Day21 | 98.5 % | 97.9 % | 84.6 % | 84.6 % | 18.25 % | 21.6 % | 13.9 % | 11.6 % |

**Supplementary Table S2.** Flow cytometry analysis of Treg under starved and non-starved culture conditions before Treg-CM collection.

|  | **CD45+** | | **CD45+**  **CD4+** | | **CD45+**  **CD4+**  **CD127dim/-CD25+** | | **CD45+**  **CD4+**  **CD127dim/-**  **CD25+**  **FOXP3+** | |
| --- | --- | --- | --- | --- | --- | --- | --- | --- |
| **Starvation** | - | + | - | + | - | + | - | + |
| **Donor 1** | 93.3 % | 93.3 % | 87.7 % | 87.7 % | 53.5 % | 55.3 % | 50.7 % | 37.0 % |
| **Donor 2** | 94.2 % | 94.2 % | 95.7% | 95.7% | 45.2 % | 46.3 % | 58.1 % | 50.3 % |
| **Donor 3** | 89.2 % | 89.2 % | 91.0 % | 91.0 % | 57.5 % | 55.9% | 28.2 % | 57.3 % |
| **Donor 4** | 90.5 % | 90.5 % | 96.1 % | 96.1 % | 41.0 % | 41.7 % | 66.0 % | 46.2 % |
| **Donor 5** | 99.2 % | 99.1 % | 86.6% | 87.7 % | 39.4 % | 37.7 % | 86.5 % | 91.0 % |
| **Donor 6** | 92.0 % | 89.9 % | 92.0 % | 65.8 % | 41.8 % | 51.7 % | 78.7 % | 72.3 % |

**Supplementary Table S3.** List of primers used for quantitative PCR analysis.

| **Gene Symbol** | **Gene name** | **Assay ID** |
| --- | --- | --- |
| *GAPDH* | glyceraldehyde-3-phosphate dehydrogenase | Hs99999905-m1 |
| *RUNX2* | runt related transcription factor 2 | Hs01047973-m1 |
| *SP7* | Sp7 transcription factor | Hs01866874-s1 |
| *COL1α2* | collagen type I alpha 2 chain | Hs00164099-m1 |
| *SPP1* | secreted phosphoprotein 1 | Hs00959010-m1 |
| *BGALP* | bone gamma-carboxyglutamate protein | Hs000609452-g1 |
| *TGFB1* | transforming growth factor beta 1 | Hs00998133-m1 |
| *ALP* | alkaline phosphatase. liver/bone/kidney | Hs01029144-m1 |

**Supplementary Table S4.** Absolute concentrations of analytes assessed by multiplex immunoassay from supernatants of BMSC treated with different conditions for 7 days.

| **Chemokine** | | | | |
| --- | --- | --- | --- | --- |
|  | | | | |
| Marker | Group | Average Level (pg/ml) | SEM | P-value |
| IL-8 | Day 0 | 102.25 | 21.61 | 0.01 |
|  | OM | 195.42 | 56.01 |  |
|  | Non-CM | 174.44 | 34.03 |  |
|  | Treg-CM | 153.40 | 25.77 |  |
| IP-10 | Day 0 | 0.00 | 0.00 | 0.44 |
|  | OM | 0.00 | 0.00 |  |
|  | Non-CM | 0.00 | 0.00 |  |
|  | Treg-CM | 19.21 | 19.21 |  |
| Eotaxin | Day 0 | 0.00 | 0.00 | >0.99 |
|  | OM | 0.00 | 0.00 |  |
|  | Non-CM | 0.00 | 0.00 |  |
|  | Treg-CM | 0.00 | 0.00 |  |
| MCP-1 | Day 0 | 205.08 | 20.50 | 0.27 |
|  | OM | 286.82 | 47.65 |  |
|  | Non-CM | 185.76 | 40.48 |  |
|  | Treg-CM | 199.82 | 30.72 |  |
| MIP-1α | Day 0 | 0.00 | 0.00 | <0.0001 |
|  | OM | 0.06 | 0.06 |  |
|  | Non-CM | 0.00 | 0.00 |  |
|  | Treg-CM | 1.81 | 0.02 |  |
| MIP-1β | Day 0 | 0.00 | 0.00 | >0.99 |
|  | OM | 0.00 | 0.00 |  |
|  | Non-CM | 0.00 | 0.00 |  |
|  | Treg-CM | 0.00 | 0.00 |  |
| RANTES | Day 0 | 0.00 | 0.00 | >0.99 |
|  | OM | 0.00 | 0.00 |  |
|  | Non-CM | 0.00 | 0.00 |  |
|  | Treg-CM | 0.00 | 0.00 |  |
| **Growth Factor** | | | | |
| Marker | Group | Average Level (pg/ml) | SEM | P-value |
| FGF-basic | Day 0 | 0.00 | 0.00 | 0.44 |
|  | OM | 0.00 | 0.00 |  |
|  | Non-CM | 0.00 | 0.00 |  |
|  | Treg-CM | 0.52 | 0.52 |  |
| G-CSF | Day 0 | 0.89 | 0.45 | 0.01 |
|  | OM | 1.71 | 1.11 |  |
|  | Non-CM | 2.75 | 1.78 |  |
|  | Treg-CM | 47.40 | 6.46 |  |
| GM-CSF | Day 0 | 0.00 | 0.00 | >0.99 |
|  | OM | 0.00 | 0.00 |  |
|  | Non-CM | 0.00 | 0.00 |  |
|  | Treg-CM | 0.00 | 0.00 |  |
| PDGF-BB | Day 0 | 0.00 | 0.00 | >0.99 |
|  | OM | 0.00 | 0.00 |  |
|  | Non-CM | 0.00 | 0.00 |  |
|  | Treg-CM | 0.00 | 0.00 |  |
| VEGF | Day 0 | 891.50 | 31.11 | 0.26 |
|  | OM | 671.67 | 138.91 |  |
|  | Non-CM | 532.13 | 140.07 |  |
|  | Treg-CM | 572.17 | 153.49 |  |
| **Pro-inflammatory / Anti-inflammatory Marker** | | | | |
| Marker | Group | Average Level (pg/ml) | SEM | P-value |
| IL-1β | Day 0 | 0.57 | 0.21 | 0.30 |
|  | OM | 0.41 | 0.24 |  |
|  | Non-CM | 0.06 | 0.06 |  |
|  | Treg-CM | 0.45 | 0.17 |  |
| IL-2 | Day 0 | 0.39 | 0.23 | <0.0001 |
|  | OM | 1.71 | 0.72 |  |
|  | Non-CM | 15.66 | 0.08 |  |
|  | Treg-CM | 9.89 | 0.32 |  |
| IL-6 | Day 0 | 478.39 | 51.73 | 0.00 |
|  | OM | 212.41 | 22.39 |  |
|  | Non-CM | 181.32 | 28.99 |  |
|  | Treg-CM | 269.13 | 50.85 |  |
| IL-7 | Day 0 | 0.00 | 0.00 | 0.44 |
|  | OM | 0.00 | 0.00 |  |
|  | Non-CM | 0.00 | 0.00 |  |
|  | Treg-CM | 0.39 | 0.39 |  |
| IL-9 | Day 0 | 0.00 | 0.00 | >0.99 |
|  | OM | 0.00 | 0.00 |  |
|  | Non-CM | 0.00 | 0.00 |  |
|  | Treg-CM | 0.00 | 0.00 |  |
| IL-12 | Day 0 | 0.03 | 0.02 | 0.18 |
|  | OM | 0.23 | 0.00 |  |
|  | Non-CM | 0.03 | 0.02 |  |
|  | Treg-CM | 0.28 | 0.17 |  |
| IL-15 | Day 0 | 3.76 | 1.88 | 0.56 |
|  | OM | 9.21 | 6.59 |  |
|  | Non-CM | 0.00 | 0.00 |  |
|  | Treg-CM | 5.77 | 5.77 |  |
| IL-17A | Day 0 | 0.00 | 0.00 | 0.44 |
|  | OM | 0.05 | 0.05 |  |
|  | Non-CM | 0.00 | 0.00 |  |
|  | Treg-CM | 0.00 | 0.00 |  |
| IFN-ɣ | Day 0 | 69.78 | 8.62 | 0.62 |
|  | OM | 81.84 | 20.40 |  |
|  | Non-CM | 54.22 | 16.49 |  |
|  | Treg-CM | 63.00 | 10.16 |  |
| TNF-α | Day 0 | 0.00 | 0.00 | >0.99 |
|  | OM | 0.00 | 0.00 |  |
|  | Non-CM | 0.00 | 0.00 |  |
|  | Treg-CM | 0.00 | 0.00 |  |
| IL-1ra | Day 0 | 15.06 | 7.53 | 0.55 |
|  | OM | 23.49 | 8.96 |  |
|  | Non-CM | 8.57 | 4.28 |  |
|  | Treg-CM | 13.61 | 6.80 |  |
| IL-4 | Day 0 | 0.84 | 0.18 | 0.01 |
|  | OM | 0.84 | 0.18 |  |
|  | Non-CM | 0.17 | 0.14 |  |
|  | Treg-CM | 0.69 | 0.09 |  |
| IL-5 | Day 0 | 35.42 | 5.44 | 0.20 |
|  | OM | 23.43 | 7.47 |  |
|  | Non-CM | 18.53 | 3.20 |  |
|  | Treg-CM | 34.16 | 6.67 |  |
| IL-10 | Day 0 | 0.00 | 0.00 | 0.01 |
|  | OM | 1.65 | 0.00 |  |
|  | Non-CM | 0.03 | 0.03 |  |
|  | Treg-CM | 0.58 | 0.54 |  |
| IL-13 | Day 0 | 0.00 | 0.00 | >0.99 |
|  | OM | 0.00 | 0.00 |  |
|  | Non-CM | 0.00 | 0.00 |  |
|  | Treg-CM | 0.00 | 0.00 |  |

**Supplementary Table S5.** Functional classification of the unique proteins identified in Treg-CM based on UniProt. Data were retrieved from the UniProt database (UniProt, 2025).

| Gene Symbol | Protein Name | Abundance | Function |
| --- | --- | --- | --- |
| H2BC12 | Histone H2B type 1-K | 24.6814 | A core component of the nucleosome. crucial for chromosomal stability. DNA packaging. and gene regulation. |
| CFH | Complement factor H | 24.5904 | A glycoprotein that prevents complement activation and amplification on cell surfaces. |
| PLG | Plasminogen | 24.5664 | A zymogen of plasmin involved in fibrin degradation. tissue remodeling. and tumor invasion. |
| C4A | Complement C4-A | 24.4245 | Essential for the propagation of the classical complement pathway. |
| GAPDH | Glyceraldehyde-3-phosphate dehydrogenase | 24.4179 | Involved in glycolysis and regulates cytoskeletal and nuclear events. as well as immune responses. |
| ITIH2 | Inter-alpha-trypsin inhibitor heavy chain H2 | 24.1979 | Similar to ITIH1. modulating the localization. synthesis. and degradation of hyaluronan. |
| ITIH1 | Inter-alpha-trypsin inhibitor heavy chain H1 | 24.0208 | Modulates the localization. synthesis. and degradation of hyaluronan. |
| APOB | Apolipoprotein B-100 | 23.9932 | Acts as a recognition signal for the cellular binding and internalization of low-density lipoprotein (LDL). |
| MSN | Moesin | 23.8642 | Connects the actin cytoskeleton to the plasma membrane. regulating cell structure and immunity. |
| RNF10 | E3 ubiquitin-protein ligase RNF10 | 23.6036 | Catalyzes the monoubiquitination of 40S ribosomal proteins. |
| IGHG4 | Immunoglobulin heavy constant gamma 4 | 23.3712 | Represents the constant region of immunoglobulin heavy chains. |
| C4BPA | C4b-binding protein alpha chain | 23.0238 | Controls the classical pathway of complement activation. |
| TRAPPC10 | Trafficking protein particle complex subunit 10 | 22.6835 | A subunit of the transport protein particle II. involved in Golgi trafficking. |
| ACTC1 | Actin. alpha cardiac muscle 1 | 22.4203 | A key structural protein involved in cell motility. |
| HSP90AB1 | Heat shock protein HSP 90-beta | 22.2375 | A chaperone protein involved in protein maturation. stabilization. transcription. and immune response. |
| HSP90AA1 | Heat shock protein HSP 90-alpha | 22.1785 | A chaperone protein involved in protein maturation. stabilization. transcription. and immune response. |
| F2 | Prothrombin | 21.9848 | A precursor to thrombin involved in blood homeostasis. inflammation. and wound healing. |
| IGHV4-39 | Immunoglobulin heavy variable 4-39 | 21.9214 | V region of the variable domain of immunoglobulin heavy chains. crucial for antigen recognition. |
| WDR1 | WD repeat-containing protein 1 | 21.7972 | Modulates actin dynamics. cytokinesis. cell migration. and epithelial organization. |
| IGHV3-7 | Immunoglobulin heavy variable 3-7 | 21.7509 | V region of the variable domain of immunoglobulin heavy chains. crucial for antigen recognition. |
| CORO1A | Coronin-1A | 20.9838 | A component of the cytoskeleton of highly motile cells. |
| PKM | Pyruvate kinase PKM | 20.908 | Involved in glycolysis. |
| C9 | Complement component C9 | 20.8804 | A component of the membrane attack complex. involved in the immune response. |
| LDHA | L-lactate dehydrogenase A chain | 20.657 | Catalyzes the conversion of pyruvate to lactate and vice versa. important for cellular metabolism. |
| HLA-A | HLA class I histocompatibility antigen. A alpha chain | 20.471 | An antigen-presenting major histocompatibility complex class I (MHCI) molecule. |
| ARHGDIB | Rho GDP-dissociation inhibitor 2 | 20.4114 | Regulates GDP/GTP exchange in Rho proteins and the reorganization of the actin cytoskeleton. |
| C7 | Complement component C7 | 20.3686 | A component of the membrane attack complex. involved in both innate and adaptive immune responses. |
| TUBA1B | Tubulin alpha-1B chain | 20.3036 | A major component of microtubules. |
| PROS1 | Vitamin K-dependent protein S | 19.8837 | An anticoagulant plasma protein. |
| C1S | Complement C1s subcomponent | 19.8727 | A serine protease involved in the complement system. |
| H2AX | Histone H2AX | 19.7317 | Replaces H2A in nucleosomes. involved in DNA accessibility. repair. and checkpoint-mediated arrest. |
| IGHV1-69D | Immunoglobulin heavy variable 1-69D | 19.7268 | V region of the variable domain of immunoglobulin heavy chains. crucial for antigen recognition. |
| SPOCK2 | Testican-2 | 19.7215 | Involved in neurogenesis and binds calcium. |
| NPM1 | Nucleophosmin | 19.6032 | Regulates ribosome biogenesis. centrosome duplication. and cellular growth. |
| EEF2 | Elongation factor 2 | 19.5757 | Catalyzes GTP-dependent translocation during translation elongation. |
| HNRNPA1 | Heterogeneous nuclear ribonucleoprotein A1 | 19.3974 | Involved in pre-mRNA packaging. mRNA transport. splicing. and translation. |
| C1R | Complement C1r subcomponent | 19.3141 | A serine protease involved in the complement system. |
| CLU | Clusterin | 19.208 | Prevents aggregation of non-native proteins and regulates apoptosis and proteasomal degradation. |
| ARPC1B | Actin-related protein 2/3 complex subunit 1B | 18.9342 | A component of the Arp2/3 complex. involved in actin polymerization. |
| RPL22 | Large ribosomal subunit protein eL22 | 18.8502 | Another component of the large ribosomal subunit. involved in protein synthesis. |
| C8B | Complement component C8 beta chain | 18.8362 | A component of the membrane attack complex. involved in the immune response. |
| RACK1 | Small ribosomal subunit protein RACK1 | 18.8126 | Regulates PKC. AKT. and IGF1R signaling pathways. as well as ribosome function and cellular processes. |
| C6 | Complement component C6 | 18.7899 | A component of the membrane attack complex. involved in the immune response. |
| RPL15 | Large ribosomal subunit protein eL15 | 18.6621 | A component of the large ribosomal subunit. involved in protein synthesis. |
| CAPZA1 | F-actin-capping protein subunit alpha-1 | 18.6359 | Binds to the end of actin filaments. stabilizing dynactin structure. |
| PGK1 | Phosphoglycerate kinase 1 | 18.2338 | Catalyzes ATP production in glycolysis. |
| ARPC2 | Actin-related protein 2/3 complex subunit 2 | 18.0587 | Another component of the Arp2/3 complex. involved in actin polymerization and cell motility. |
| TNFRSF8 | Tumor necrosis factor receptor superfamily member 8 | 18.0339 | Regulates cellular growth. lymphoblast transformation. and activates NF-kappa-B signaling. |
| EZR | Ezrin | 17.895 | Connects the cytoskeleton to the membrane and is essential for microvilli formation. |
| CD3E | T-cell surface glycoprotein CD3 epsilon chain | 17.5669 | Crucial for T-cell activation and development through TCR-CD3 signaling. |
| IGHV5-10-1 | Immunoglobulin heavy variable 5-10-1 | 17.4835 | V region of the variable domain of immunoglobulin heavy chains. crucial for antigen recognition. |
| FABP5 | Fatty acid-binding protein 5 | 17.3844 | Transports fatty acids and lipids. regulates nuclear receptors. and modulates inflammation. |
| CD82 | CD82 antigen | 17.3811 | Involved in receptor signaling. adhesion. migration. and protein trafficking. |
| GDI2 | Rab GDP dissociation inhibitor beta | 17.3045 | Prevents GDP-GTP exchange in Rab proteins. regulating membrane trafficking and ciliogenesis. |
| F12 | Coagulation factor XII | 17.3011 | Initiates blood coagulation. fibrinolysis. and generates bradykinin and angiotensin. |
| PTPRC | Receptor-type tyrosine-protein phosphatase C | 17.2722 | Required for T cell activation and involved in the immune response. |
| BSG | Basigin | 17.2395 | Regulates retinal growth and cellular proliferation. acting as a receptor for pathogens. |
| SERBP1 | SERPINE1 mRNA-binding protein 1 | 17.1684 | Regulates ribosome hibernation and mRNA stability. |
| HNRNPA2B1 | Heterogeneous nuclear ribonucleoproteins A2/B1 | 17.1543 | Involved in transcription. mRNA processing. transport. translation. and stability. |
| TSPAN14 | Tetraspanin-14 | 17.1388 | Involved in ADAM10 maturation. trafficking to the cell surface. and substrate specificity. |
| DPYSL2 | Dihydropyrimidinase-related protein 2 | 17.1254 | Modulates neuronal development. axon growth. guidance. and cytoskeleton remodeling. |
| FDPS | Farnesyl pyrophosphate synthase | 16.9864 | Facilitates farnesyl diphosphate formation and is involved in protein modification. |
| FCN3 | Ficolin-3 | 16.9193 | Activates the lectin complement pathway. involved in innate immunity. |
| C4BPB | C4b-binding protein beta chain | 16.9069 | Regulates complement activation and facilitates immune complex clearance. |
| PON1 | Serum paraoxonase/arylesterase 1 | 16.8573 | Hydrolyzes toxic organophosphates and protects lipoproteins from oxidative modification. |
| SNRNP70 | U1 small nuclear ribonucleoprotein 70 kDa | 16.7981 | A component of the spliceosome. crucial for recognizing pre-mRNA splice sites. |
| P0DOX3 | Immunoglobulin delta heavy chain | 16.7741 | involvend in adaptive immunity |
| HSPA4 | Heat shock 70 kDa protein 4 | 16.7248 | An ATP-dependent protein folding chaperone. |
| RPL7A | Large ribosomal subunit protein eL8 | 16.6415 | A component of the large ribosomal subunit. involved in protein synthesis. |
| RPL35 | Large ribosomal subunit protein uL29 | 16.62 | A component of the large ribosomal subunit. involved in protein synthesis. |
| RPS15A | Small ribosomal subunit protein uS8 | 16.5689 | A small ribosomal subunit component. crucial for small ribosomal subunit assembly and erythropoiesis. |
| TRA2B | Transformer-2 protein homolog beta | 16.4917 | Regulates pre-mRNA splicing. including exon inclusion or exclusion. |
| NCL | Nucleolin | 16.4307 | Facilitates chromatin decondensation and is involved in ribosome assembly and pre-rRNA transcription. |
| ACTN4 | Alpha-actinin-4 | 16.3678 | An F-actin cross-linking protein involved in trafficking and T cell activation. |
| SELE | E-selectin | 16.3666 | A cell surface glycoprotein involved in immunoadhesion. |
| HSP90B1 | Endoplasmin | 16.2951 | A chaperone protein involved in the folding. processing. and transport of secreted proteins. |
| DPP3 | Dipeptidyl peptidase 3 | 16.2871 | Cleaves and degrades bioactive peptides. |
| XPO1 | Exportin-1 | 16.1769 | Mediates the nuclear export of proteins and RNAs. |
| HNRNPU | Heterogeneous nuclear ribonucleoprotein U | 16.138 | Involved in transcription. RNA processing. chromatin organization. and the cell cycle. |
| LCP1 | Plastin-2 | 16.1308 | An actin-binding protein involved in T cell activation and IL2RA/CD25 and CD69 surface expression. |
| CLSTN1 | Calsyntenin-1 | 16.0742 | A postsynaptic adhesion molecule facilitates synapse formation and cargo transport. |
| RPS7 | Small ribosomal subunit protein eS7 | 16.0208 | A small ribosomal subunit component required for RNA maturation and small ribosomal subunit assembly. |
| FASN | Fatty acid synthase | 16.0148 | Catalyzes de novo fatty acid synthesis from acetyl-CoA and malonyl-CoA. |
| SERPINB1 | Leukocyte elastase inhibitor | 15.9881 | A neutrophil serine protease inhibitor that regulates the innate immune system and inflammation. |
| RBMXL2 | RNA-binding motif protein. X-linked-like-2 | 15.8583 | An RNA-binding protein involved in mRNA splicing. |
| TXNRD1 | Thioredoxin reductase 1. cytoplasmic | 15.4623 | Regulates redox reactions. reduces thioredoxin. and modulates cellular growth and differentiation. |
| RPL28 | Large ribosomal subunit protein eL28 | 15.3284 | A component of the large ribosomal subunit. involved in protein synthesis. |
